# Positive Attribute Framing Increases COVID-19 Booster Vaccine Intention for Unfamiliar Vaccines

**DOI:** 10.3390/vaccines10060962

**Published:** 2022-06-16

**Authors:** Kirsten Barnes, Ben Colagiuri

**Affiliations:** School of Psychology, The University of Sydney, Brennan MacCallum Building (A18), Manning Rd, Camperdown, Sydney, NSW 2006, Australia; ben.colagiuri@sydney.edu.au

**Keywords:** vaccination, COVID-19, vaccine hesitancy, vaccine intention, positive framing, attribute framing

## Abstract

Positive framing has been proposed as an intervention to increase COVID-19 vaccination intentions. However, available research has examined fictitious or unfamiliar treatments. This pre-registered study (aspredicted#78369) compared the effect of standard negatively framed EU patient information leaflets (PILs), with new positively framed PILs, on booster intentions (measured pre- and post-intervention) for AstraZeneca, Pfizer, and Moderna COVID-19 vaccines. A representative sample of 1222 UK-based adults was randomised to one of six groups in a factorial design with framing (Positive vs. Negative) and vaccine familiarity (same (as previous), familiar, unfamiliar) as factors. The benefit of positive framing was hypothesised to be strongest for the least familiar vaccine (Moderna). Framing was moderated by familiarity, where only the unfamiliar vaccine showed a benefit of positive relative to negative Framing. Framing and familiarity also interacted with baseline Intention with the effect of framing on the unfamiliar vaccine especially pronounced at low baseline Intent. Conversely, standard negative framing appeared to increase intentions for familiar vaccines at low baseline intent. Findings provide important evidence that positive framing could improve vaccine uptake globally when switches or new developments require individuals to receive less familiar vaccines. Positive framing of familiar vaccines, however, should be treated with caution until better understood.

## 1. Introduction

With vaccine efficacy for Severe Acute Respiratory Syndrome Coronavirus 2 (COVID-19) waning over time [1,2] and reduced for emerging variants [3,4], many countries are accelerating their COVID-19 booster programmes [5]. However, vaccine availability does not necessarily translate to vaccine acceptance [6], with the World Health Organization (WHO) recognising vaccine hesitancy as a global health threat [7]. Side effect apprehension is a primary factor driving hesitancy [8], with 90% of COVID-19 vaccine refusers fearing side effects more than COVID-19 itself [9], and side effect severity from initial doses associated with booster hesitancy [10]. Reducing perceptions of side effects appears vital for increasing booster acceptance and reducing the global burden of COVID-19.

The WHO [11] has suggested that the framing of vaccine-relevant information (e.g., [12]) could provide a method of reducing negative perceptions. Positive attribute framing, where side effect information is framed in terms of the inverse incidence rate (e.g., “60% will ***not*** get a sore arm”) as opposed to typical negative framing with the standard incidence rate (e.g., “40% will get a sore arm”), could be particularly useful for combatting COVID-19 vaccine hesitancy. First, it is directly applicable to side effects. Second, informed consent is maintained due to statistical consistency across frames [13]. Third, there is preliminary evidence that positive attribute framing can improve vaccination attitudes in other settings. For example, one study on the influenza vaccine found positive attribute framing (hereafter positive framing) reduced the expectation and experience of side effects, increased perceived protection from influenza, and reduced distortions in the perception of side effect risk [14], with results replicated for other vaccine types [15].

The handful of studies examining framing on COVID-19 vaccine intention have produced mixed results [16,17,18,19]. However, those studies focused on vaccine-naïve individuals, did not employ attribute framing, and did not concern booster intentions. Further, those studies also either used fictitious COVID-19 vaccines [16,17] or did not name specific approved COVID-19 vaccines [18,19]. As such, participants either had limited knowledge of, or investment in, the framed vaccines. As the pandemic has progressed, media discourse [20,21] combined with direct and socially-observed experience of COVID-19 side effects [22], means it is essential to understand whether positive framing is effective for real-world vaccines where prior knowledge and experience exists. This is particularly important because there is reason to believe that prior knowledge regarding a given COVID-19 vaccine may moderate the strength of any positive framing effect.

Research has shown that greater relevance or belief in a treatment or issue decreases the efficacy of different forms of framing [23,24], including positive framing on perceptions of hypothetical vaccines [23,25]. The effect of positive framing on vaccine intention may therefore be limited to less familiar vaccines. Even if so, positive framing may still hold utility. New composition changes to COVID-19 booster vaccines have been recommended [26] and are currently being developed [27] to protect against emerging variants. Further, many booster programmes (e.g., in the United Kingdom) require switches from an experienced vaccine (e.g., AstraZeneca Vaxzevria) to a less familiar one (e.g., Moderna Spikevax). Positive framing may therefore be beneficial for increasing uptake for novel vaccines and switches to less familiar vaccines. Yet, because research on positive framing has largely focused on fictitious medications and patient scenarios [23,25,28,29,30,31,32], the extent to which familiarity moderates the effect of positive framing is currently unclear. Therefore, to understand the extent to which positive framing could be deployed to improve global vaccine uptake to combat COVID-19, it is critical to test the efficacy of positive framing for genuine familiar and unfamiliar vaccines.

In this pre-registered study, positive attribute framing was applied to side effects from genuine manufacturer Patient Information Leaflets (PILs) for the AstraZeneca, Pfizer, and Moderna vaccines and compared to standard negative wording. Participants were randomised to read positive or negatively framed PILs for the same vaccine they had previously received (either AstraZeneca/Pfizer), a familiar vaccine in the UK context (Pfizer/AstraZeneca), or an unfamiliar vaccine (Moderna). It was hypothesized that (a) positive framing would increase booster intention, and (b) the effect of positive framing would decrease with vaccine familiarity (i.e., an interaction between framing and familiarity, with the effect of positive framing decreasing with familiarity). Following previous research [13,28,33,34,35], secondary outcome variables concerning booster side effect severity, perceived risk, and booster acceptance, as well as prevalence judgments, were explored as potential mediators of the framing effect on vaccine intention (see Supplementary Materials: S1.1).

## 2. Methods and Materials

Ethical approval was obtained from the University of Sydney Human Research Ethics Committee (reference, 2021/792). The research was pre-registered (aspredicted #78369).

### 2.1. Participants

Participants (*N* = 1222) were recruited from the UK via Pureprofile, an ISO-certified panel provider, between 27 October to 8 November 2021. Inclusion criteria were: 18 years of age or older; currently residing in the UK; self-reported English fluency; previously received two doses of the Pfizer or two doses of the AstraZeneca COVID-19 vaccines; not received a COVID-19 booster vaccine; and no known medical reason (e.g., allergy) prohibiting administration of the COVID-19 vaccines framed. Participants were reimbursed £3.50 for a ~15-min survey.

### 2.2. Design

**Figure 1:**
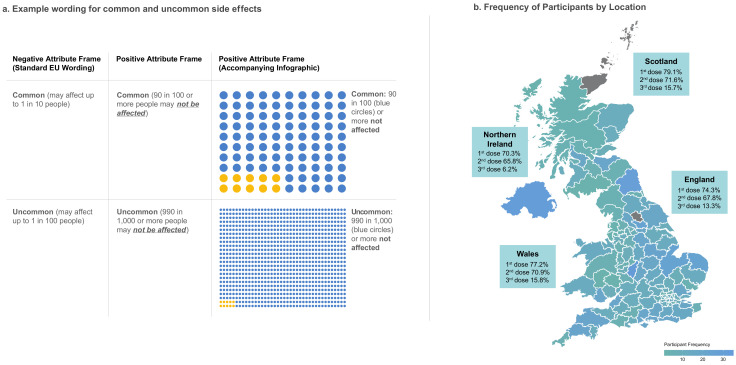
(**a**)**.** Positive and negative wording used to frame common and uncommon side effects (wording for all prevalence categories can be found in Supplementary Materials S1.3); (**b**). frequency of participants from each postal area of the UK plotted against the vaccination rates reported by the UK government on 3 November (the final week of data collection).

A between-subjects 2(framing) x 3(familiarity) factorial design was employed with participants stratified by previous vaccine type (i.e., AstraZeneca vs. Pfizer) and randomised to one of the six conditions. Those receiving negative framing viewed genuine manufacturer PILs for either the AstraZeneca, Pfizer, or Moderna vaccine. Those receiving positive framing viewed the same PILs but containing the inverse side effect incident rate (i.e., the number ***not*** affected). Figure 1 provides example wording for common and uncommon side effects (full wording and PILs presented in Supplementary Materials S1.2 and S1.3).

To manipulate familiarity, participants were randomised to view PILs from the following conditions: ‘Same’ (PIL for the COVID-19 vaccine previously received: AstraZeneca-AstraZeneca|Pfizer-Pfizer); ‘Familiar’ (PIL for a common vaccine not previously received: Pfizer-AstraZeneca|AstraZeneca-Pfizer); and ‘Unfamiliar’ (PIL for a less common vaccine in the UK: AstraZeneca-Moderna|Pfizer-Moderna). Familiarity was judged on UK data (22nd September 2021), where fewer Modena second doses (1.2 million) had been administered relative to the two primary vaccine types available in the UK at the time: the Pfizer and AstraZenca vaccine (19.4 and 24.0 million doses administered respectively [36]).

### 2.3. Data Collection: Primary and Secondary Outcomes

**Figure 2:**
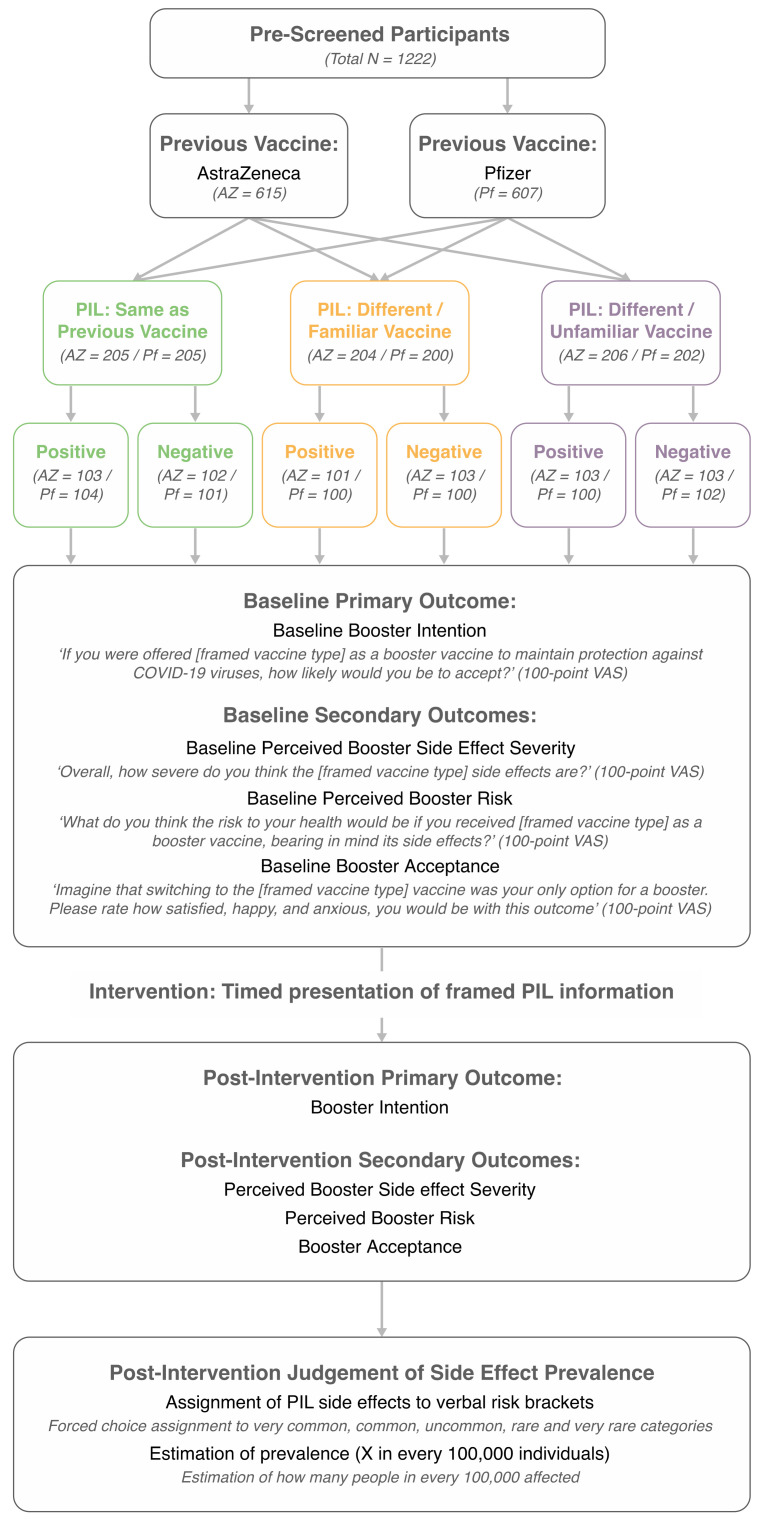
Overview of the design, including the item wording for primary and secondary outcomes. *Nb.* Satisfaction, happiness, and anxiety were rated separately as part of the booster acceptance measure. Primary and secondary outcomes employed a 100-point VAS with the following anchors: booster intention (*‘definitely would not accept vaccine’* vs. *‘definitely would accept vaccine’)*; booster side effect severity (*‘not at all severe’* vs. *‘extremely severe’*); perceived booster risk (*‘extremely low risk’* vs. *‘extremely high risk’*); and booster acceptance (*‘not at all’* vs. *‘extremely’*).

Primary and secondary outcomes were collected pre- and post-intervention. The primary outcome was the participant’s intention to receive a booster vaccine (booster intention). Secondary outcomes (measured as potential mediators and presented as Supplementary Materials: S1.1) were: booster side effect severity; perceived risk; and booster acceptance. Outcome wording is presented in Figure 2. Familiarity with the side effects of the AstraZeneca, Pfizer, and Moderna vaccines (100-point VAS) were additionally assessed pre-intervention to determine whether side effect knowledge corresponded with the predetermined factorial categories of vaccine familiarity (i.e., same > familiar > unfamiliar). At the end of the study, all participants made post-intervention judgements of side effect prevalence in order to assess general inaccuracies in side effect representation post-framing (see Supplementary Materials S1.1).

### 2.4. Data Collection and Quotas

Cross-sectional data were collected online via Qualtrics, with the survey accessible to personal computer, tablet, and smartphone. The ‘force response’ option was used to ensure complete cases for all outcome variables. Participants completed the survey in one sitting and could not return to the study URL.

Data collection occurred single-blind. Participants were aware of the framed information, but not the presence of the other conditions. Stratified randomisation to the six experimental groups occurred via the inbuilt Qualtrics randomisation function. Quotas were set to limit data collection to 600 participants from each prior vaccine stratum, with 100 from each randomised to one of the six experimental conditions. Because Qualtrics tallies quotas on survey completion (not accounting for participants in the experimental pipeline), the final sample contained 22 more participants than projected. No statistical analysis took place until after exclusions had been made and all quotas closed.

### 2.5. Procedure

After pre-screening and consent, participants completed demographic items and identified which COVID-19 vaccine they had previously received (AstraZeneca or Pfizer). Stratified randomisation was subsequently performed. Participants responded to items concerning months since their last COVID-19 vaccine, familiarity with side effects of the three framed vaccines, and provided pre-intervention ratings for primary and secondary outcomes (see Figure 2) for each vaccine type (AstraZeneca, Pfizer, Moderna). Responses made to the vaccine type that matched the experimental condition to which the participant had been assigned were employed as baseline measures. Responses to all other vaccine types were recorded for use in a concurrent, but separate, pre-registered study (see: aspredicted.org/8e6af.pdf accessed on 28 October 2021).

PILs were then displayed for 2 minutes, using a timer embedded in the survey. Participants could not proceed until this time had elapsed. Post-intervention primary and secondary outcomes were subsequently recorded. Finally, participants categorised 14 side effects reported in their PIL into verbal prevalence categories and provided frequency estimates. On completion, all participants were provided with an electronic debrief for download and URLs to the UK government landing page where the original PILs for the vaccines employed in the study could be found.

Several additional items concerning general COVID-19 booster intentions, perceived risk of previous COVID-19 vaccines, specific COVD-19 vaccination side effects, and general perceptions of COVID-19 and COVID-19 vaccinations, were included in the survey prior to the intervention for use in a separate pre-registered study (see: aspredicted.org/8e6af.pdf accessed on 28 October 2021).

### 2.6. Survey Materials: Descriptive Variables

#### 2.6.1. Demographic Information

Participants responded to items concerning their age, gender, ethnicity, highest level of education and employment status, and geographic region (postal area code).

#### 2.6.2. Previous Exposure to COVID-19

Items were employed to capture personal exposure to COVID-19, as well as exposure through close friends and family. Item wording (To your knowledge, are you, or have you been, infected with COVID-19?/To your knowledge, have any of your close family members or friends been infected with COVID-19?) was taken from the WHO ‘Behavioural and Social Drivers of Vaccination Guidebook’ [11].

#### 2.6.3. Previous COVID-19 Vaccination History 

Previous COVID-19 vaccine (Pfizer/AstraZeneca) was recorded as a forced-choice option. Participants indicated the number of months since their last COVID-19 vaccine, and whether their most severe side effects occurred with their first dose, second dose, whether they were equal across doses, or not experienced at all (forced-choice).

#### 2.6.4. Familiarity with COVID-19 Vaccine Side Effects

For the three framed vaccines, participants were asked to rate their “familiarity with the potential side effects” on a 100-point VAS (anchors: ’not at all familiar’/’extremely familiar’) pre-intervention. Those who had not heard of the vaccine were asked to check a separate ‘not heard of vaccine’ box but received a score of zero (‘not at all familiar’). This response-type was used to exclude inconsistent responders (see Supplementary Materials S1.4).

### 2.7. Survey Materials: Inferential Variables

#### 2.7.1. Primary and Secondary Outcomes

Wording of the four variables employed as primary and secondary outcomes are presented in Figure 2, with wording adapted from previous research [35]. Booster acceptance, satisfaction, happiness, and anxiety, were rated separately. Where the vaccine type was the same as that previously received by the participant, wording was changed from ‘switching to’ to ‘continuing with’: e.g., “Imagine that continuing with the [framed vaccine type] vaccine was your only option for a booster. Please rate how satisfied, happy, and anxious, you would be with this outcome”. Pre-intervention ratings were given for all three vaccines, while post-intervention ratings were recorded only for the vaccine outlined in the assigned PIL.

#### 2.7.2. Post-Intervention Judgement of Side Effect Prevalence

Fourteen side effects were presented from each PIL (see Supplementary Materials S1.5). Eleven side effects were associated with discrete prevalence categories. Three were presented in the PILs as of ‘unknown prevalence’. Participants were required to classify side effects into verbal prevalence categories, “based on the information that you read, how common do you think [side effect] is?” (forced-choice: very common, common, uncommon, rare, very rare). They also provided frequency estimates: “In 100,000 people, how many do you think would experience [side effect] if they received a [framed vaccine name] booster vaccine?” (free-response, limited to numbers at up to 10 decimal places).

### 2.8. Patient Information Leaflets (PILs)

Genuine PILs for the AstraZeneca, Pfizer, and Moderna vaccine were abridged to include the manufacturer’s description of each vaccine; what it is used for; and critically, the possible side effects resulting from administration. Side effects were retained in their original form and order. For both negative and positive framing, standard EU verbal prevalence categories were presented as published by the manufacturer (i.e., very common, common, uncommon, rare, very rare, and not known). For negative framing, wording of assigned frequency bands was identical to that of the manufacturer. However, this was inverted for positive framing to stress the number of individuals not affected (e.g., “common (90 in 100 or more people may *not be affected*)”). As multi-modal forms of side effect presentation (e.g., written, pictorial, verbal) may elicit larger framing effects [13], and numeracy is less likely to moderate the effect of attribute framing for graphical presentations [37], positively framed PILs additionally included a graphical representation of side effect risk to enhance the intervention.

### 2.9. Statistical Analysis and Sample Size

Pre-registered analyses for primary and secondary outcomes were 2(framing) x 3(familiarity) factorial ANCOVAs, with the baseline measure as the covariate. However, baseline measures were found to systematically differ by familiarity (see results). To avoid violating the assumptions of ANCOVA [38], we addressed this by extending the model to include the interactions between the covariate and manipulated variables (Framing and Familiarity), as has previously been recommended [39]. Pre-specified orthogonal contrasts for familiarity were: Contrast1 (Same vs. Other [Familiar and Unfamiliar combined]) and Contrast2 (Familiar vs. Unfamiliar). Pre-registered subsidiary analysis of the primary outcome concerned realistic vaccine switches occurring as part of the UK’s booster programme. Those without medical exemption who received AstraZeneca will be required to switch to Pfizer or Moderna (2(framing) x 2(familiar vs. unfamiliar) ANCOVA), and those who received Pfizer may be required to switch to Moderna (one-way ANCOVA restricted to the Unfamiliar Vaccine). Pre-registered analysis of secondary predictors are included in Supplementary Materials S1.1.

Sample size (estimated *N* = 1200) was calculated based on an a priori power analysis (95% power, alpha = 0.05, effect size *f*^2^ = 0.02) for a separate study run concurrently that contained more predictor variables (*N* = 9) and required more power than the current study (see pre-registration form). An a priori effect size for attribute framing was additionally derived from previous research (average effect size *r* = 0.175) [13], with 491 participants required for the ANCOVA model, providing reassurance that the projected sample size provided ample power to detect an effect of framing.

## 3. Results

### 3.1. Sample 

A total of 1896 eligible participants provided electronic consent and 1459 completed the study (data was automatically deleted for those who closed their browser mid-study). A further 237 completing participants were removed based on pre-registered quality control criteria (see Supplementary Materials S1.4). Analysis was performed on data from the remaining 1222 participants.

### 3.2. Descriptive Statistics 

**Figure 3:**
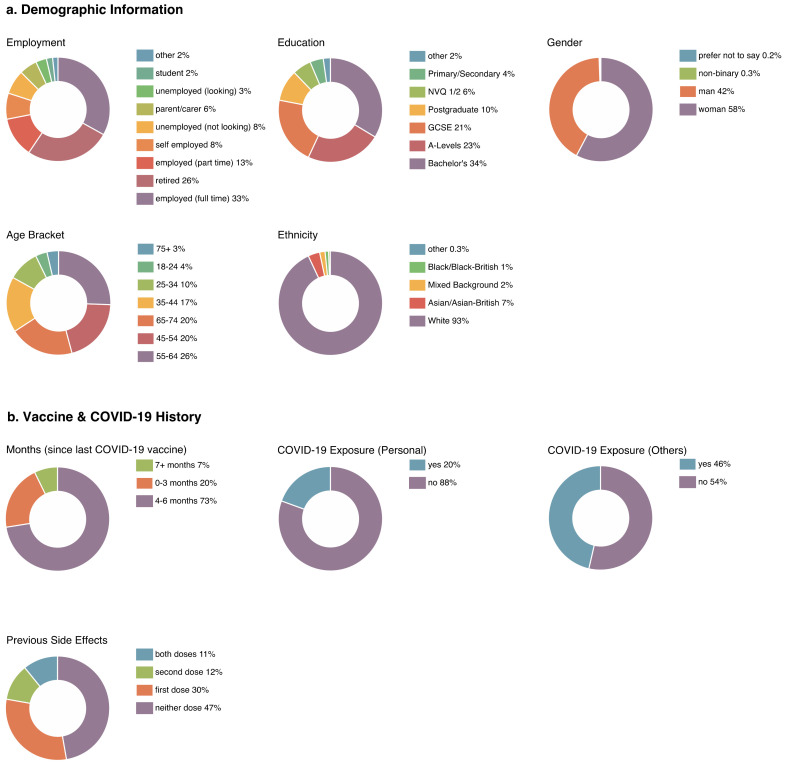
Descriptive statistics regarding sample demographics, as well as vaccine and COVID-19 history of the full sample (*N* = 1222).

Participants were 52.5 years of age on average (range = 18–95) and resided across most postal areas in the UK, with the largest proportion from London district (*N* = 53), Birmingham (*N* = 35), and Belfast (*N* = 34). Only Harrogate, and the Orkney and Shetland Islands were not represented. Information regarding participant location can be found in Figure 1b. Descriptive statistics regarding demographic information and vaccine and COVID-19 history for the full sample can be found in Figure 3a,b respectively. Demographic information by Condition can be found in Table 1, and information regarding vaccine and COVID-19 history by Condition in Table 2.

### 3.3. Primary Analysis

#### 3.3.1. Knowledge of Vaccine Side Effects Mirrors Categorical Levels of the Familiarity Factor

**Figure 4:**
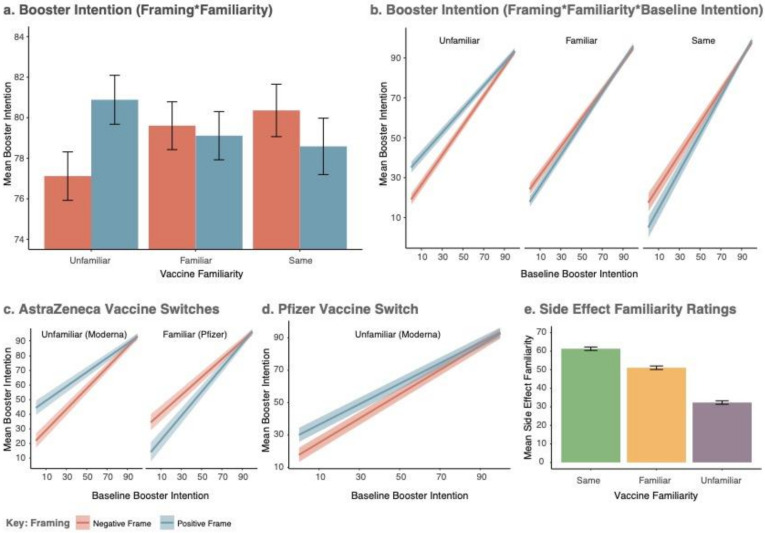
Model estimated mean differences in the primary outcome (Booster Intention), depicted for the whole sample ((**a**,**b**); sample size by condition presented in Table 1), and for realistic switches occurring as part of the UK booster programme ((**c**,**d**); AstraZeneca/Unfamiliar *N* = 206, AstraZeneca/Familiar *N* = 204, Pfizer/Unfamiliar *N* = 202, sample size by condition presented in Figure 2). (**e**) presents data demonstrating that side effect familiarity ratings scaled with the factorial levels of vaccine familiarity (within-subjects, full sample *N* = 1222). All error bars represent ± 1SEM.

To determine whether side effect familiarity corresponded with the predetermined factorial categories of familiarity, a within-subjects one-way ANOVA (with Greenhouse–Geisser correction) was run on pre-intervention side effect familiarity ratings. A robust main effect of familiarity was observed (*F*(1.86, 2272.18) = 659.17, *p* < 0.001, *η_p_^2^* = 0.35). Awareness of side effects increased with familiarity; being higher for the same vs. familiar vaccine (*t*(1221) = 14.11, *p* < 0.001, Cohen’s *d_z_* = 0.40), and for the familiar vs. unfamiliar vaccine (*t*(1221) = 23.97, *p* < 0.001, Cohen’s *d_z_* = 0.69). Mean differences are presented in Figure 4e.

#### 3.3.2. Baseline Vaccine Intention

A 2(framing) x 3(familiarity) ANOVA was conducted on baseline booster intention to assess the presence of between-group differences. Ratings were anticipated to be high across conditions (see pre-registration), which was confirmed in the present sample (*M* = 78.36 (/100-point VAS), *SD* = 31.65; range: 0–100). However, an unanticipated significant effect of familiarity was observed (*F*(2, 1216) = 49.51, *p* < 0.0001, *η_p_^2^* = 0.075). This effect reached statistical significance for the orthogonal contrast comparing the same vs. other (i.e., combined familiar and unfamiliar) vaccine types (*F*(1, 1216) = 96.51, *p* < 0.0001, *η_p_^2^* = 0.074), but not for the familiar vs. unfamiliar comparison (*F*(1, 1216) = 2.42, *p* = 0.120, *η_p_^2^* = 0.002), indicating higher intentions for previously experienced vaccines.

#### 3.3.3. Effect of Framing and Familiarity on the Intention to Be Vaccinated

The anticipated framing x familiarity interaction was observed (*F*(2, 1210) = 10.75, *p* < 0.0001, *η_p_^2^* = 0.018), where framing interacted with Contrast1 (Same vs. Other: *F*(1, 1210) = 5.07, *p* = 0.025, *η_p_^2^* = 0.004) and Contrast2 (Familiar vs. Unfamiliar: *F*(1, 1210) = 16.46, *p* = 0.0001, *η_p_^2^* = 0.013). As demonstrated in Figure 4a, this pattern of results was driven by positive framing increasing booster intention for the unfamiliar vaccine. However, this was superseded by a three-way interaction with baseline booster intention (*F*(2, 1210) = 7.65, *p* = 0.0005, *η_p_^2^* = 0.013), represented at both contrasts (baseline x framing x Contrast1: *F*(1, 1210) = 4.39, *p* = 0.036, *η_p_^2^* = 0.004 | Baseline x Framing x Contrast2: *F*(1, 1210) = 11.19, *p* = 0.0008, *η_p_^2^* = 0.009). Figure 4b depicts this interaction. Positive Framing had limited efficacy at high levels of baseline Intention across conditions but took effect for the unfamiliar vaccine when the model-estimated baseline intention scores were lower than ~80/100. At very low levels of baseline intention (i.e., VAS = 0), model predicted booster intention post-intervention increased from *M* = 19.09 (*SEM* = 2.76, 95% CIs [13.68, 24.50]) for the negative frame, to *M* = 35.11 (*SEM* = 2.77, 95% CIs [29.68, 40.50]) for the positive frame. Full model output is included in Supplementary Materials S1.6. We note that the same framing x familiarity interaction was observed in the planned but invalid model excluding the interaction between manipulated factors and covariate (see Supplementary Materials S1.7).

### 3.4. The Effect of Framing on Vaccine Switches

#### 3.4.1. Interactions between Previous Vaccine Type and Experimental Factors

Previous vaccine type (AstraZeneca/Pfizer) was entered as a factor in the ANCOVA model above to check for interactions with framing, familiarity, and baseline booster intention. While there was a main effect of previous vaccine type (*F*(1, 1198) = 6.36, *p* = 0.012, *η_p_^2^* = 0.005), with those receiving AstraZeneca reporting increased booster intention (*M* = 80.83, *SEM*= 0.74, 95% CIs [79.37, 82.29]) compared to Pfizer (*M* = 78.01, *SEM*= 0.74, 95% CIs [76.57, 79.46]), there were no two- or three-way interactions with framing or familiarity (all *ps* > 0.05; see Supplementary Materials S1.8).

#### 3.4.2. Previous Vaccine: AstraZeneca

A framing x familiarity x baseline intention interaction was observed on booster intention (*F*(1, 402) = 11.38, *p* = 0.0008, *η_p_^2^* = 0.028) among those previously receiving AstraZeneca (*N* = 410). As demonstrated in Figure 4c, in the case of the unfamiliar vaccine (Moderna), booster intention was increased in the positive frame at low levels of baseline booster intention. However, the inverse of this pattern was observed for the familiar vaccine (Pfizer), where positive framing decreased booster Intention at low levels of baseline intention (full model; Supplementary Materials S1.9).

#### 3.4.3. Previous Vaccine: Pfizer

Among those who previously received the Pfizer vaccine (*N* = 202), there was a main effect of framing (*F*(1, 198) = 3.98, *p* = 0.048, *η_p_^2^* = 0.020), but no statistically significant baseline booster intention x framing interaction (*F*(1, 198) = 1.80, *p* = 0.181, *η_p_^2^* = 0.009). However, as demonstrated in Figure 4d, slopes for the positive and negative frame converged at high levels of baseline booster intention (full model; Supplementary Materials S1.10).

## 4. Discussion

Message framing has been suggested as a potential intervention to increase COVID-19 vaccine uptake [11]. We examined the effect of positive and negative attribute framing of side effect information on booster intentions for three genuine COVID-19 vaccines varying in familiarity. Positive framing successfully increased booster intention for the unfamiliar vaccine (i.e., Moderna), but reduced intention for the vaccine previously received, as well as for a switch to Pfizer among those previously receiving AstraZeneca. In all cases, effects were strongest at low baseline booster intentions. Increasing booster acceptance among those with low intentions is of substantial importance in protecting against infection from, and transmission of, COVID-19 viruses. Critically, our data suggest that any intervention intending to employ attribute framing should be carefully tailored to match the framed information (positive vs. negative wording) with vaccine familiarity. Specifically, positive framing appears to have significant potential in situations where a novel vaccine or composition changes are being introduced [26,27]. By contrast, positive framing may actually be harmful when the vaccine is familiar.

The effect of positive attribute framing on booster intentions for the unfamiliar vaccine is consistent with medical decision-making research. In these studies, framed information has typically been presented regarding fictitious medications and patient scenarios [23,25,28,29,30,31,32]. When employing real treatments, data has been collected from samples where participants were completely [14,40] or largely [41] naïve to the framed treatment, or where prior treatment experience was not assessed [15,42]. The current data thereby provide new insights into the effect of framing. Under conditions directly relevant to the COVID-19 pandemic (i.e., for real vaccines, at high levels of public involvement), the benefit of positive attribute framing was found to wane, or be reversed, as familiarity and prior experience with the framed vaccine increased. As such, calls for all PILs to employ positive framing as standard (e.g., [28,43]) appear premature. Instead, negative framing, the standard form for communicating side effect information within the European Union, appears beneficial when treatments are well known.

The reduced efficacy of positive framing with increased vaccine familiarity could be explained by a current theory of attribute framing that posits an interaction between familiarity (a manifestation of psychological distance) and the valence of the message surrounding a given attribute or event (e.g., the experience of vaccine side effects). At closer psychological distances (e.g., for vaccines that are more familiar and more likely to be received), negatively framed information has been shown to be more persuasive [44]. Further experimental research is needed to test this theory, while considering alternative explanations, such as the role of potential backfire effects in persuasive or corrective messaging, which participants with low intent may have considered positive framing to be, particularly when the vaccine was familiar or more likely to be received. Such effects are known to impact attitudes surrounding the COVID-19 pandemic [45] and have been demonstrated to lower intentions for other vaccine types at high levels of concern [46]. However, when assessed in conjunction, current results highlight the fact that any intervention that strives to apply positive framing across all vaccine types, irrespective of familiarity, should be treated with caution.

The psychological mechanisms underlying the effect of positive framing on booster intentions remain unclear. We measured secondary variables as potential mediators. However, results did not mirror those obtained for the primary outcome—booster intention (see Supplementary Materials S1.1). While changes in secondary variables (side effect severity and booster acceptance) were observed with framing, post-hoc analysis (see Supplementary Materials S1.11) plotting the familiarity x framing x baseline interaction for those who had high vs. low baseline booster intent, suggested that these framing-induced changes largely occurred among those with high vaccine intention at baseline. As these participants also showed a limited effect of framing on their behavioural intention to be vaccinated, the relationship between booster intention and our secondary predictors appears orthogonal. An investigation of other factors combined with qualitative research may be better positioned to identify the driving factors behind the effect of framing on COVID-19 booster intentions. Further, we note that, consistent with previous reports [35,47,48,49,50], prevalence judgements were poor (<~35% accuracy). This appeared exacerbated among those receiving positive framing, but again did not differ by familiarity. As side effects differed by PIL, the current study was designed only to test for general inaccuracies in side effect representation and not systematic over- or under-estimation. Experimental studies are therefore needed to assess precisely how any inaccuracies associated with positively framed COVID-19 vaccine information manifest.

The primary strength of the present study is the application of attribute framing to real COVID-19 vaccine information. The PILs employed here are displayed on government and NHS websites in the UK, forming a primary official source of information regarding COVID-19 vaccination. Our findings therefore have real-world implications, demonstrating that the wording of PILs can directly impact the intention to receive a booster vaccine among individuals for whom this decision is both directly relevant and imminent. There are of course some limitations worth noting including the collection of cross-sectional data that limits an assessment of the durability of the framing effect, as well as a sample located within a single country. Given global differences in booster policy, cross-cultural replication of results is required to ensure results are not contextually limited to the UK. While vaccine intention has been demonstrated to be a strong predictor of vaccine uptake (e.g., [51,52,53]), including for COVID-19 vaccination [54], we do not assume that the two are synonymous (e.g., [55]). While beyond the scope of the present study, we recommend that future research incorporate longitudinal designs where the rate of conversion from intention to vaccine uptake can be tracked, as well as consider the effect of framing on those under 18 years of age. Further, present results are specific to booster intentions among those already vaccinated. While side effect apprehension has been associated with hesitancy regarding COVID-19 vaccination [8] and booster vaccination [10], whether a similar pattern of results would hold among those who have never been vaccinated is unknown.

In summary, the present study demonstrates that the ability of positive framing to successfully increase booster intention for genuine COVID-19 vaccines is critically moderated by the familiarity of that vaccine. Positive framing can improve vaccine intention for unfamiliar vaccines, but may actually decrease intentions for familiar vaccines. The data therefore provide novel insights into the benefits of positive framing for COVID-19 vaccines and beyond. As such, we recommend that if positive attribute framing is to be employed, close attention must be paid to the type of treatment being framed as well as the likely recipients of the framed information. Importantly, in the context of the current COVID-19 pandemic, positive framing appears capable of improving the uptake of COVID-19 vaccines when switches or new developments require individuals to receive unfamiliar vaccines, as is the case for many booster vaccine programmes globally.

## Figures and Tables

**Table 1:** Descriptive statistics (frequency counts) regarding demographics information for each experimental condition.

	Same Positive *N =* 207	Same Negative *N =* 203	Familiar Positive *N =* 201	Familiar Negative *N =* 203	Unfamiliar Positive *N =* 203	Unfamiliar Negative *N =* 205
***Employment***						
Employed full-time	68	63	70	61	72	73
Employed part-time	16	28	21	36	23	29
Self employed	16	19	14	17	11	21
Unemployed (looking)	8	6	5	9	6	4
Unemployed (not looking)/long-term sick or disabled	17	16	14	16	14	15
Parent/Carer	10	13	10	10	17	10
Student	3	4	2	6	4	5
Retired	66	52	58	47	52	46
Other	3	2	7	1	4	2
***Education***						
Primary/Secondary (no qualifications)	11	15	10	7	7	3
GCSE (or equivalent)	36	44	39	48	44	44
NVQ 1/2	16	10	10	13	10	11
A/AS-Levels (or equivalent)	53	50	44	46	45	47
Bachelor’s degree ;(or equivalent)	65	66	76	61	74	71
Post-Graduate	21	14	17	21	21	27
Other	5	4	5	7	2	2
***Gender***						
Woman	117	122	106	123	122	116
Man	90	81	93	79	81	87
Non-binary	0	0	1	1	0	1
Prefer not to say (other)	0	0	1	0	0	1
***Age bracket (years)***						
18–24	8	8	3	10	7	11
25–34	17	23	20	18	18	20
35–44	42	27	30	41	32	39
45–54	34	49	38	38	46	41
55–64	52	49	59	52	53	50
65–74	48	38	41	39	40	39
75+	6	9	10	5	7	5
***Ethnicity***						
Asian or Asian British ^a^	5	10	6	3	9	11
Black or Black British ^b^	5	4	3	1	1	2
Mixed background ^c^	4	2	5	4	1	4
Other ^d^	3	0	0	0	1	0
White ^e^	190	187	187	195	191	188

^a^ Asian or Asian British includes those who identified as Bangladeshi, Indian, Pakistani, Chinese, or as being from ‘any other Asian background’. ^b^ Black or Black British includes those who identified as Black Carribean or Black African. ^c^ Mixed background includes those who identified as White and Black African, White and Black Caribbean, White and Asian, or as being from ‘any other Mixed background’. ^d^ Other includes ‘other ethinic group’ or ‘other (not stated)’. ^e^ White includes those who identified as White British, White Irish, or as being from ‘any other White background’.

**Table 2:** Descriptive statistics (frequency counts) regarding vaccine and COVID-19 history for each experimental condition.

	Same Positive *N =* 207	Same Negative *N =* 203	Familiar Positive *N =* 201	Familiar Negative *N =* 203	Unfamiliar Positive *N =* 203	Unfamiliar Negative *N =* 205
***Months (since last COVID-19 vaccination)***						
0–3	41	44	38	48	34	42
4–6	156	148	147	143	153	141
7+	10	11	16	12	16	22
***COVID-19 Exposure: Personal infection***						
Yes	24	23	27	26	22	26
No	183	180	174	177	181	179
***COVID-19 Exposure: Significant others***						
Yes	108	86	87	98	92	94
No	99	117	114	105	111	111
***Previous Side Effects***						
Yes (First Dose)	65	61	57	61	66	62
Yes (Second Dose)	23	26	20	28	20	24
Yes (Both Doses)	23	17	22	21	21	26
No	96	99	102	93	96	93

## Data Availability

The code and raw data necessary to replicate the reported analysis is available through the Open Science Framework repository (means/SEMs needed to reproduce the analysis Figures are included with the raw data): https://osf.io/d5cvn/?view_only=500c83d90bb9416796e464108ad2fc41 (accessed on 20 January 2022).

## Supplementary Materials

The following supporting information can be downloaded at: https://www.mdpi.com/article/10.3390/vaccines10060962/s1, S1: Supplementary Materials.

## References

[1] Andrews N., Tessier E., Stowe J., Gower C., Kirsebom F., Simmons R., Gallagher E., Chand M., Brown K., Ladhani S.N. (2021). Vaccine effectiveness and duration of protection of Comirnaty, Vaxzevria and Spikevax against mild and severe COVID-19 in the UK. medRxiv.

[2] Goldberg Y., Mandel M., Bar-On Y.M., Bodenheimer O., Freedman L., Haas E.J., Milo R., Alroy-Preis S., Ash N., Huppert A. (2021). Waning immunity of the BNT162b2 vaccine: A nationwide study from Israel. medRxiv.

[3] Cele S., Jackson L., Khan K., Khoury D.S., Moyo-Gwete T., Tegally H., Scheepers C., Amoako D., Karim F., Bernstein M. (2021). SARS-CoV-2 Omicron has extensive but incomplete escape of Pfizer BNT162b2 elicited neutralization and requires ACE2 for infection. medRxiv.

[4] Basile K., Rockett R.J., McPhie K., Fennell M., Johnson-Mackinnon J., Agius J.E., Fong W., Rahman H., Ko D., Donavan L. (2021). Improved neutralization of the SARS-CoV-2 Omicron variant after Pfizer-BioNTech BNT162b2 COVID-19 vaccine boosting. bioRxiv.

[5] Mahase E. (2021). Covid-19 booster vaccines: What we know and who’s doing what. BMJ.

[6] MacDonald N.E., Eskola J., Liang X., Chaudhuri M., Dube E., Gellin B., Goldstein S., Larson H., Manzo M.L., Reingold A. (2015). Vaccine Hesitancy: Definition, Scope and Determinants. Vaccine.

[7] World Health Organization (WHO) (2019). Ten Threats to Global Health in 2019. https://www.who.int/news-room/spotlight/ten-threats-to-global-health-in-2019.

[8] Arce J.S.S., Warren S.S., Meriggi N.F., Scacco A., McMurry N., Voors M., Syunyaev G., Malik A.A., Aboutajdine S., Adeojo O. (2021). COVID-19 vaccine acceptance and hesitancy in low- and middle-income countries. Nat. Med..

[9] (2021). Why Won’t Americans Get Vaccinated?. https://today.yougov.com/topics/politics/articles-reports/2021/07/15/why-wont-americans-get-vaccinated-poll-data.

[10] Rzymski P., Poniedziałek B., Fal A. (2021). Willingness to Receive the Booster COVID-19 Vaccine Dose in Poland. Vaccines.

[11] World Health Organization (WHO) Data for Action: Achieving High Uptake of COVID-19 vaccines: Gathering and Using Data on the Behavioural and Social Drivers of Vaccination: A Guidebook for Immunization Programmes and Implementing Partners: Interim guidance, 1 April 2021. https://apps.who.int/iris/handle/10665/340645.

[12] Levin I.P., Schneider S., Gaeth G.J. (1998). All Frames Are Not Created Equal: A Typology and Critical Analysis of Framing Effects. Organ. Behav. Hum. Decis. Process..

[13] Barnes K., Faasse K., Geers A.L., Helfer S.G., Sharpe L., Colloca L., Colagiuri B. (2019). Can Positive Framing Reduce Nocebo Side Effects? Current Evidence and Recommendation for Future Research. Front. Pharmacol..

[14] O’Connor A.M., Pennie R.A., Dales R.E. (1996). Framing effects on expectations, decisions, and side effects experienced: The case of influenza immunization. J. Clin. Epidemiol..

[15] Bigman C.A., Cappella J.N., Hornik R.C. (2010). Effective or ineffective: Attribute framing and the human papillomavirus (HPV) vaccine. Patient Educ. Couns..

[16] Sudharsanan N., Favaretti C., Hachaturyan V., Bärnighausen T., Vandormael A. (2021). Effects of Side-Effect Risk Framing Strategies on COVID-19 Vaccine Intentions in the United States and the United Kingdom: A Randomized Controlled Trial. medRxiv.

[17] Chen T., Dai M., Xia S., Zhou Y. (2021). Do Messages Matter? Investigating the Combined Effects of Framing, Outcome Uncertainty, and Number Format on COVID-19 Vaccination Attitudes and Intention. Health Commun..

[18] Huang Y., Liu W. (2021). Promoting COVID-19 Vaccination: The Interplay of Message Framing, Psychological Uncertainty, and Public Agency as a Message Source. Sci. Commun..

[19] Betta S., Castellini G., Acampora M., Barello S. (2022). The Effect of Message Framing on COVID-19 Vaccination Intentions among the Younger Age Population Groups: Results from an Experimental Study in the Italian Context. Vaccines.

[20] Lentzen M.-P., Huebenthal V., Kaiser R., Kreppel M., Zoeller J.E., Zirk M. (2022). A retrospective analysis of social media posts pertaining to COVID-19 vaccination side effects. Vaccine.

[21] Jamison A.M., Broniatowski D.A., Dredze M., Wood-Doughty Z., Khan D., Quinn S.C. (2020). Vaccine-related advertising in the Facebook Ad Archive. Vaccine.

[22] Ndwandwe D., Wiysonge C.S. (2021). COVID-19 vaccines. Curr. Opin. Immunol..

[23] Donovan R.J., Jalleh G. (2000). Positive versus Negative Framing of a Hypothetical Infant Immunization: The Influence of Involvement. Health Educ. Behav..

[24] Benjamin D., Por H.-H., Budescu D. (2016). Climate Change Versus Global Warming: Who Is Susceptible to the Framing of Climate Change?. Environ. Behav..

[25] Haydarov R., Gordon J.C. (2015). Effect of combining attribute and goal framing within messages to change vaccination behavior. J. Commun. Healthc..

[26] (2022). Interim Statement on COVID-19 Vaccines in the Context of the Circulation of the Omicron SARS-CoV-2 Variant from the WHO Technical Advisory Group on COVID-19 Vaccine Composition (TAG-CO-VAC) [Internet] [World Health Organization]. https://www.who.int/news/item/11-01-2022-interim-statement-on-covid-19-vaccines-in-the-context-of-the-circulation-of-the-omicron-sars-cov-2-variant-from-the-who-technical-advisory-group-on-covid-19-vaccine-composition.

[27] (2022). Pfizer plans to manufacture up to 100 million doses of omicron-specific vaccine by spring. The Washington Post.

[28] Webster R.K., Rubin G.J. (2020). The Effect of Positively Framing Side-Effect Risk in Two Different Formats on Side-Effect Expectations, Informed Consent and Credibility: A Randomised Trial of 16- to 75-Year-Olds in England. Drug Saf..

[29] Krishnamurthy P., Carter P., Blair E. (2001). Attribute Framing and Goal Framing Effects in Health Decisions. Organ. Behav. Hum. Decis. Process..

[30] Levin I.P., Schnittjer S.K., Thee S.L. (1988). Information framing effects in social and personal decisions. J. Exp. Soc. Psychol..

[31] Zimmermann C., Baldo C., Molino A. (2000). Framing of outcome and probability of recurrence: Breast cancer patients’ choice of adjuvant chemotherapy (ACT) in hypothetical patient scenarios. Breast Cancer Res. Treat..

[32] Marteau T.M. (1989). Framing of information: Its influence upon decisions of doctors and patients. Br. J. Soc. Psychol..

[33] Webster R.K., Rubin G.J. (2021). Predicting Expectations of Side-Effects for Those Which Are Warned Versus Not Warned About in Patient Information Leaflets. Ann. Behav. Med..

[34] Herber O.R., Gies V., Schwappach D., Thürmann P., Wilm S. (2014). Patient information leaflets: Informing or frightening? A focus group study exploring patients’ emotional reactions and subsequent behavior towards package leaflets of commonly prescribed medications in family practices. BMC Fam. Pract..

[35] Berry D.C., Raynor D.K., Knapp P. (2003). Communicating risk of medication side effects: An empirical evaluation of EU recommended terminology. Psychol. Health Med..

[36] (2021). Research and Analysis: Coronavirus Vaccine—Weekly Summary of Yellow Card Reporting [Internet] [gov.uk]. https://www.gov.uk/government/publications/coronavirus-covid-19-vaccine-adverse-reactions/coronavirus-vaccine-summary-of-yellow-card-reporting.

[37] Kreiner H., Gamliel E. (2017). Are highly numerate individuals invulnerable to attribute framing bias? Comparing numerically and graphically represented attribute framing. Eur. J. Soc. Psychol..

[38] Myers J.L., Well A.D. (2003). Research Design and Statistical Analysis.

[39] Cardinal R.N., Aitken M.R.F. (2006). ANOVA for the Behavioural Sciences Researcher.

[40] Llewellyn-Thomas H.A., McGreal M.J., Thiel E.C. (1995). Cancer Patients’ Decision Making and Trial-entry Preferences: The Effects of “Framing” Information about Short-term Toxicity and Long-term Survival. Med. Decis. Mak..

[41] Ferguson E., Gallagher L. (2007). Message framing with respect to decisions about vaccination: The roles of frame valence, frame method and perceived risk. Br. J. Psychol..

[42] Jasper J.D., Goel R., Einarson A., Gallo M., Koren G. (2001). Effects of framing on teratogenic risk perception in pregnant women. Lancet.

[43] Webster R.K., Weinman J., Rubin G.J. (2019). Explaining all without causing unnecessary harm: Is there scope for positively framing medical risk information?. Patient Educ. Couns..

[44] Freling T.H., Vincent L.H., Henard D.H. (2014). When not to accentuate the positive: Re-examining valence effects in attribute framing. Organ. Behav. Hum. Decis. Process..

[45] Dan V., Dixon G.N. (2021). Fighting the Infodemic on Two Fronts: Reducing False Beliefs Without Increasing Polarization. Sci. Commun..

[46] Nyhan B., Reifler J. (2015). Does correcting myths about the flu vaccine work? An experimental evaluation of the effects of corrective information. Vaccine.

[47] Webster R.K., Weinman J., Rubin G.J. (2017). People’s Understanding of Verbal Risk Descriptors in Patient Information Leaflets: A Cross-Sectional National Survey of 18- to 65-Year-Olds in England. Drug Saf..

[48] Berry D.C., Knapp P.R., Raynor T. (2002). Is 15 per cent very common? Informing people about the risks of medication side effects. Int. J. Pharm. Pract..

[49] Berry D.C., Raynor D., Knapp P., Bersellini E. (2003). Patients’ Understanding of Risk Associated with Medication Use. Drug Saf..

[50] Knapp P., Raynor D.K., Woolf E., Gardner P., Carrigan N., McMillan B., Knapp P. (2009). Communicating the Risk of Side Effects to Patients. Drug Saf..

[51] Gerend M.A., Shepherd J.E. (2012). Predicting human papillomavirus vaccine uptake in young adult women: Comparing the health belief model and theory of planned behavior. Ann. Behav. Med..

[52] Lehmann B.A., Ruiter R.A.C., Chapman G., Kok G. (2014). The intention to get vaccinated against influenza and actual vaccination uptake of Dutch healthcare personnel. Vaccine.

[53] Juraskova I., Bari R.A., O’Brien M.T., McCaffery K.J. (2011). HPV Vaccine Promotion: Does Referring to Both Cervical Cancer and Genital Warts Affect Intended and Actual Vaccination Behavior?. Women’s Health Issues.

[54] Jensen U., Ayers S., Koskan A. (2021). Video-based messages to reduce COVID-19 vaccine hesitancy and nudge uptake. PsyArXiv.

[55] Sheeran P. (2002). Intention—Behavior Relations: A Conceptual and Empirical Review. Eur. Rev. Soc. Psychol..

