# Supplementary material for: Positive Attribute Framing Increases COVID-19 Booster Vaccine Intention for Unfamiliar Vaccines
Source: Vaccines (Basel). 2022 Jun 16;10(6):962. doi: 10.3390/vaccines10060962 (PMC9229566; doi:10.3390/vaccines10060962)

S1: Supporting Materials

Contents

S1.1: Analysis of Secondary Variables **2**

S1.2: Wording used for the Positive and Negative Attribute Frame **16**

S1.3: Patient Information Leaflets (PILs) **17**

S1.4: Pre-analysis data cleaning **24**

S1.5: Side effects presented from each PIL **25**

S1.6: Full statistical model for the primary outcome **29**

S1.7: Pre-registered ANCOVA (without interactions between factors and covariate) **31**

S1.8: The effect of positive framing on vaccine switches **33**

S1.9: Vaccine Switch - AstraZeneca to Pfizer and Moderna **34**

S1.10: Vaccine Switch - Pfizer to Moderna **35**

S1.11: Exploratory Analysis **37**

S1.1: Analysis of Secondary Variables

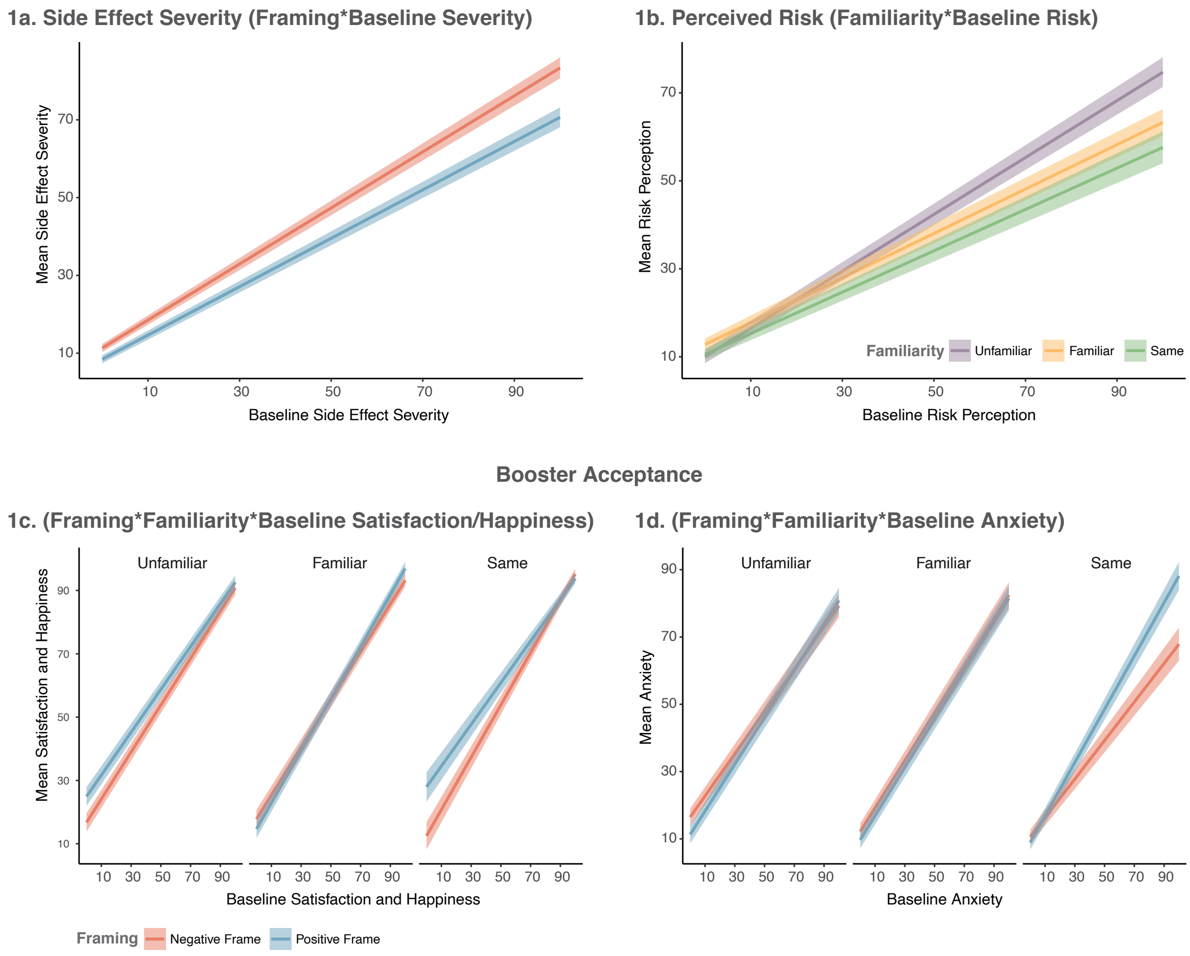

Supplementary Figure S1: Mean values for secondary outcome measures, where 1a depicts model-estimated mean differences regarding side effect severity ratings, 1b perceptions of vaccine risk, 1c combined satisfaction and happiness associated with the framed vaccine, and 1d anxiety surrounding the framed vaccine. All error bars represent +/- 1SEM.

S1.1.1: Booster Side Effect Severity

Summary

Perceived side effect severity associated with the vaccines was measured pre- and post-manipulation. A 2(Framing) * 3(Familiarity) ANCOVA, including interactions with the covariate (Baseline Side Effect Severity), revealed a main effect of Framing ($F(1, 1210)=3.92$, $p=.048$, *np2*=.003), and a Baseline Severity by Framing interaction ($F(1, 1210)=4.52$, $p=.034$, *np2*=.004). There were no other two- or three-way interactions (all $ps>.35$).

As depicted in Supplementary Figure 1a, reading about side effects in any frame increased the perception of their severity (i.e., post-manipulation model estimated means are above zero, for all levels of the baseline covariate). However, Positive Framing resulted in a lower perception of side effect severity, which was strongest when side effects were perceived as severe at baseline.

Booster Side Effect Severity: Statistical Models

- sesev_fv_t2 (perceived side effect severity associated with the framed vaccine post-manipulation)
- sesev_fv_t1 (perceived side effect severity associated with the framed vaccine at baseline)

Main Effects Model

	Sum Sq	Df	F value	Pr(>F)
(Intercept)	57189.5779	1	183.2956	0.0000
sesev_fv_t1	263557.1698	1	844.7145	0.0000
Framing	1222.8893	1	3.9194	0.0480
Familiarity	40.5057	2	0.0649	0.9372
sesev_fv_t1:Framing	1411.1856	1	4.5229	0.0336
sesev_fv_t1:Familiarity	654.8262	2	1.0494	0.3505
Framing:Familiarity	157.2217	2	0.2520	0.7773
sesev_fv_t1:Framing:Familiarity	314.8565	2	0.5046	0.6039
Residuals	377528.9467	1210	NA	NA

	eta.sq	eta.sq.part
sesev_fv_t1	0.3935	0.4111
Framing	0.0018	0.0032
Familiarity	0.0001	0.0001
sesev_fv_t1:Framing	0.0021	0.0037
sesev_fv_t1:Familiarity	0.0010	0.0017
Framing:Familiarity	0.0002	0.0004
sesev_fv_t1:Framing:Familiarity	0.0005	0.0008

Model with pre-registered contrasts for Vaccine Familiarity

	Sum Sq	Df	F value	Pr(>F)
(Intercept)	57189.5779	1	183.2956	0.0000
sesev_fv_t1	263557.1698	1	844.7145	0.0000
Framing	1222.8893	1	3.9194	0.0480
cont1	40.0779	1	0.1285	0.7201
cont2	0.5846	1	0.0019	0.9655
sesev_fv_t1:Framing	1411.1856	1	4.5229	0.0336
sesev_fv_t1:cont1	609.9385	1	1.9549	0.1623
Framing:cont1	53.6660	1	0.1720	0.6784
sesev_fv_t1:cont2	51.2097	1	0.1641	0.6855
Framing:cont2	100.9352	1	0.3235	0.5696
sesev_fv_t1:Framing:cont1	156.2693	1	0.5009	0.4793
sesev_fv_t1:Framing:cont2	152.7560	1	0.4896	0.4842
Residuals	377528.9467	1210	NA	NA

	eta.sq	eta.sq.part
sesev_fv_t1	0.3935	0.4111
Framing	0.0018	0.0032
cont1	0.0001	0.0001
cont2	0.0000	0.0000
sesev_fv_t1:Framing	0.0021	0.0037
sesev_fv_t1:cont1	0.0009	0.0016
Framing:cont1	0.0001	0.0001
sesev_fv_t1:cont2	0.0001	0.0001
Framing:cont2	0.0002	0.0003
sesev_fv_t1:Framing:cont1	0.0002	0.0004
sesev_fv_t1:Framing:cont2	0.0002	0.0004

S1.1.2: Perceived Booster Risk

Summary

Perception of risk to participant's health associated with receiving the vaccines was recorded pre- and post-manipulation. The main effect of Framing did not reach statistical significance ($F(1, 1210)$=1.04, p=.308, *np2*=.001). There was a Familiarity by Baseline Risk Perception interaction ($F(2, 1210)$=4.82, p=.008, *np2*=.008), evident in both contrasts (Baseline Risk by Contrast1: $F(1, 1210)$=4.28, p=.039, *np2*=.004 | Baseline Risk by Contrast2: $F(1, 1210)$=6.03, p=.014, *np2*=.005). No other statistically significant main effects or interactions were observed (all *ps*>.10). As depicted in Supplementary Figure 1b, there was limited difference between Familiarity conditions when the perception of risk was low at baseline. At high levels of Baseline Risk, Post-Manipulation Risk Perception was lowest for the previously experienced vaccine and highest for the Unfamiliar vaccine.

Perceived Booster Risk: Statistical Models

- serisk_fv_t2 (perceived risk associated with the framed vaccine post-manipulation)
- sesev_fv_t1 (perceived risk associated with the framed vaccine at baseline)

Main Effects Model

	Sum Sq	Df	F value	Pr(>F)
(Intercept)	81175.1304	1	193.8057	0.0000
serisk_fv_t1	215659.8866	1	514.8883	0.0000
Framing	436.2962	1	1.0417	0.3076
Familiarity	894.8706	2	1.0683	0.3439
serisk_fv_t1:Framing	21.8767	1	0.0522	0.8193
serisk_fv_t1:Familiarity	4039.4547	2	4.8221	0.0082
Framing:Familiarity	74.8987	2	0.0894	0.9145
serisk_fv_t1:Framing:Familiarity	919.5879	2	1.0978	0.3340
Residuals	506805.9582	1210	NA	NA

	eta.sq	eta.sq.part
serisk_fv_t1	0.2885	0.2985
Framing	0.0006	0.0009
Familiarity	0.0012	0.0018
serisk_fv_t1:Framing	0.0000	0.0000
serisk_fv_t1:Familiarity	0.0054	0.0079
Framing:Familiarity	0.0001	0.0001
serisk_fv_t1:Framing:Familiarity	0.0012	0.0018

Model with pre-registered contrasts for Vaccine Familiarity

	Sum Sq	Df	F value	Pr(>F)
(Intercept)	81175.1304	1	193.8057	0.0000
serisk_fv_t1	215659.8866	1	514.8883	0.0000
Framing	436.2962	1	1.0417	0.3076
cont1	109.4773	1	0.2614	0.6093
cont2	754.8287	1	1.8022	0.1797
serisk_fv_t1:Framing	21.8767	1	0.0522	0.8193
serisk_fv_t1:cont1	1791.4699	1	4.2771	0.0388
Framing:cont1	11.7394	1	0.0280	0.8671
serisk_fv_t1:cont2	2526.0142	1	6.0309	0.0142
Framing:cont2	65.7193	1	0.1569	0.6921
serisk_fv_t1:Framing:cont1	708.3207	1	1.6911	0.1937
serisk_fv_t1:Framing:cont2	267.7285	1	0.6392	0.4242
Residuals	506805.9582	1210	NA	NA

	eta.sq	eta.sq.part
serisk_fv_t1	0.2885	0.2985
Framing	0.0006	0.0009
cont1	0.0001	0.0002
cont2	0.0010	0.0015
serisk_fv_t1:Framing	0.0000	0.0000
serisk_fv_t1:cont1	0.0024	0.0035
Framing:cont1	0.0000	0.0000
serisk_fv_t1:cont2	0.0034	0.0050
Framing:cont2	0.0001	0.0001
serisk_fv_t1:Framing:cont1	0.0009	0.0014
serisk_fv_t1:Framing:cont2	0.0004	0.0005

S1.1.3: Booster Acceptance (Satisfaction, Happiness, and Anxiety)

Summary

At pre- and post-manipulation, participants were asked to imagine that the framed vaccine was their only option for a booster and to rate their Satisfaction, Happiness and Anxiety regarding this outcome. In order to reduce the data for analysis, the three variables were entered into a principal components analysis. Component loading suggested that Satisfaction and Happiness loaded onto a similar latent construct, while Anxiety was associated with another. Therefore, Satisfaction and Happiness were averaged together for the subsequent analyses, while Anxiety was treated separately. Two ANCOVAs were run on these outcomes. The first revealed a main effect of Framing (F(1, 1210)=5.66, p=.018, *np2*=.005), a two-way Framing by Familiarity interaction (F(2, 1210)=3.63, p=.027, *np2*=.006) and a three-way Baseline Satisfaction/Happiness by Framing by Familiarity interaction (F(2, 1210)=3.43, p=.033, *np2*=.006). This three-way interaction was significant for Contrast1 (Same vs. Other: F(1, 1210)=4.11, p=.043, *np2*=.003), but not Contrast2 (Familiar vs. Unfamiliar: F(1, 1210)=2.56, p=.110, *np2*=.002). As shown in Supplementary Figure 1c, for the Same and Unfamiliar vaccines, lowered satisfaction and happiness were generally associated with Negative Framing, particularly at low levels of Baseline Satisfaction/Happiness. There appeared to be no difference for the Familiar vaccine.

With respect to anxiety, there was a significant Baseline Anxiety by Framing interaction (F(1, 1210)=5.53, p=.019, *np2*=.005), but no other main effects or interactions (all *ps*>.05). As shown in Supplementary Figure 1d, Positive Framing appeared to increase Post-Manipulation Anxiety at very high levels of Baseline Anxiety, but was limited to the Same Condition (i.e., among participants who had high Baseline Anxiety regarding continuation with their previous vaccine type), with this interaction nearing significance (Anxiety by Framing by Contrast1: F(1, 1210)=3.65, p=.056, *np2*=.003).

Correlations and PCA for Framed Vaccine Side Effect Perceptions

(Satisfaction, Happiness and Anxiety)

Corrrelations between variables Variables are: switchsat_fv_t2 (satisfaction with the framed vaccine post-manipulation) switchhap_fv_t2 (happiness with the framed vaccine post-manipulation) switchanx_rev_fv_t2 (anxiety with the framed vaccine post-manipulation - variable reversed (+ve association))

```
##
##  Pearson's product-moment correlation
##
## data:  sat_dat$switchsat_fv_t2 and sat_dat$switchhap_fv_t2
## t = 78.489, df = 1220, p-value < 2.2e-16
## alternative hypothesis: true correlation is not equal to 0
## 95 percent confidence interval:
##  0.9038498 0.9224377
## sample estimates:
##       cor
## 0.9136199
```

```
##
##  Pearson's product-moment correlation
##
## data:  sat_dat$switchsat_fv_t2 and sat_dat$switchanx_rev_fv_t2
## t = 16.839, df = 1220, p-value < 2.2e-16
## alternative hypothesis: true correlation is not equal to 0
## 95 percent confidence interval:
##  0.3876229 0.4786818
## sample estimates:
##       cor
## 0.4342611
```

```
##
##  Pearson's product-moment correlation
##
## data:  sat_dat$switchhap_fv_t2 and sat_dat$switchanx_rev_fv_t2
## t = 17.55, df = 1220, p-value < 2.2e-16
## alternative hypothesis: true correlation is not equal to 0
## 95 percent confidence interval:
##  0.4030299 0.4926355
## sample estimates:
##       cor
## 0.4489607
```

PCA component loadings for post-manipulation measures

	PC1	PC2	PC3
switchsat__fv__t2	0.6135009	-0.3383353	-0.7135446
switchhap__fv__t2	0.6338972	-0.3278683	0.7004832
switchanx__rev__fv__t2	0.4709469	0.8820610	-0.0133223

PCA component loadings for pre-manipulation measures

	PC1	PC2	PC3
switchsat__fv__t1	0.6469202	-0.2877156	0.7061969
switchhap__fv__t1	0.6539002	-0.2671390	-0.7078498
switchanx__rev__fv__t1	0.3923122	0.9197045	0.0153197

Satisfaction and Happiness combined: Statistical Models

- av_sathap_t2 (average satisfaction and happiness associated with the framed vaccine post-manipulation)
- av_sathap_t1 (average satisfaction and happiness associated with the framed vaccine at baseline)

Main Effects Model

	Sum Sq	Df	F value	Pr(>F)
(Intercept)	68464.8110	1	177.3429	0.0000
av_sathap_t1	629741.7591	1	1631.2060	0.0000
Framing	2184.7699	1	5.6592	0.0175
Familiarity	1042.8219	2	1.3506	0.2595
av_sathap_t1:Framing	849.7667	1	2.2011	0.1382
av_sathap_t1:Familiarity	1420.4558	2	1.8397	0.1593
Framing:Familiarity	2803.5778	2	3.6310	0.0268
av_sathap_t1:Framing:Familiarity	2651.9911	2	3.4347	0.0325
Residuals	467131.3843	1210	NA	NA

	eta.sq	eta.sq.part
av_sathap_t1	0.5173	0.5741
Framing	0.0018	0.0047
Familiarity	0.0009	0.0022
av_sathap_t1:Framing	0.0007	0.0018
av_sathap_t1:Familiarity	0.0012	0.0030
Framing:Familiarity	0.0023	0.0060
av_sathap_t1:Framing:Familiarity	0.0022	0.0056

Model with pre-registered contrasts for Vaccine Familiarity

	Sum Sq	Df	F value	Pr(>F)
(Intercept)	68464.8110	1	177.3429	0.0000
av_sathap_t1	629741.7591	1	1631.2060	0.0000
Framing	2184.7699	1	5.6592	0.0175
cont1	89.8365	1	0.2327	0.6296
cont2	949.0046	1	2.4582	0.1172
av_sathap_t1:Framing	849.7667	1	2.2011	0.1382
av_sathap_t1:cont1	7.8678	1	0.0204	0.8865
Framing:cont1	1323.4860	1	3.4282	0.0643
av_sathap_t1:cont2	1405.2058	1	3.6399	0.0566
Framing:cont2	1461.2307	1	3.7850	0.0519
av_sathap_t1:Framing:cont1	1588.5089	1	4.1147	0.0427
av_sathap_t1:Framing:cont2	987.7901	1	2.5587	0.1100
Residuals	467131.3843	1210	NA	NA

	eta.sq	eta.sq.part
av_sathap_t1	0.5173	0.5741
Framing	0.0018	0.0047
cont1	0.0001	0.0002
cont2	0.0008	0.0020
av_sathap_t1:Framing	0.0007	0.0018
av_sathap_t1:cont1	0.0000	0.0000
Framing:cont1	0.0011	0.0028
av_sathap_t1:cont2	0.0012	0.0030
Framing:cont2	0.0012	0.0031
av_sathap_t1:Framing:cont1	0.0013	0.0034
av_sathap_t1:Framing:cont2	0.0008	0.0021

Anxiety: Statistical Models

Main Effects Model

	Sum Sq	Df	F value	Pr(>F)
(Intercept)	80477.3132	1	140.8544	0.0000
switchanx_fv_t1	584744.8174	1	1023.4421	0.0000
Framing	1449.0382	1	2.5362	0.1115
Familiarity	1743.2143	2	1.5255	0.2179
switchanx_fv_t1:Framing	3160.2594	1	5.5312	0.0188
switchanx_fv_t1:Familiarity	524.9404	2	0.4594	0.6318
Framing:Familiarity	318.5277	2	0.2787	0.7568
switchanx_fv_t1:Framing:Familiarity	2257.7873	2	1.9758	0.1391
Residuals	691334.8598	1210	NA	NA

	eta.sq	eta.sq.part
switchanx_fv_t1	0.4358	0.4582
Framing	0.0011	0.0021
Familiarity	0.0013	0.0025
switchanx_fv_t1:Framing	0.0024	0.0046
switchanx_fv_t1:Familiarity	0.0004	0.0008
Framing:Familiarity	0.0002	0.0005
switchanx_fv_t1:Framing:Familiarity	0.0017	0.0033

Model with pre-registered contrasts for Vaccine Familiarity

	Sum Sq	Df	F value	Pr(>F)
(Intercept)	80477.3132	1	140.8544	0.0000
switchanx_fv_t1	584744.8174	1	1023.4421	0.0000
Framing	1449.0382	1	2.5362	0.1115
cont1	1041.1486	1	1.8223	0.1773
cont2	788.8024	1	1.3806	0.2402
switchanx_fv_t1:Framing	3160.2594	1	5.5312	0.0188
switchanx_fv_t1:cont1	4.6664	1	0.0082	0.9280
Framing:cont1	174.0626	1	0.3047	0.5811
switchanx_fv_t1:cont2	518.5455	1	0.9076	0.3409
Framing:cont2	160.4686	1	0.2809	0.5962
switchanx_fv_t1:Framing:cont1	2086.2670	1	3.6515	0.0563
switchanx_fv_t1:Framing:cont2	152.6742	1	0.2672	0.6053
Residuals	691334.8598	1210	NA	NA

	eta.sq	eta.sq.part
switchanx_fv_t1	0.4358	0.4582
Framing	0.0011	0.0021
cont1	0.0008	0.0015
cont2	0.0006	0.0011
switchanx_fv_t1:Framing	0.0024	0.0046
switchanx_fv_t1:cont1	0.0000	0.0000
Framing:cont1	0.0001	0.0003
switchanx_fv_t1:cont2	0.0004	0.0007
Framing:cont2	0.0001	0.0002
switchanx_fv_t1:Framing:cont1	0.0016	0.0030
switchanx_fv_t1:Framing:cont2	0.0001	0.0002

S1.1.4: Judgements Regarding Side Effect Prevalence

Summary

Participants were required to classify 14 side effects from their assigned PIL (see tables in 1.4.2 below) into verbal prevalence categories, as well as provide a frequency estimate regarding how many people, in 100,000, they thought would experience each side effect if they received the framed vaccine. Eleven side effects were associated with discrete prevalence categories. Three were presented in the PILs as of 'unknown prevalence'. Raw frequency estimates of the latter were analysed separately to test whether framing alters perceptions regarding prevalence when no information is provided. For side effects with a concrete category, the percentage of side effects correctly assigned across verbal prevalence categories, and frequency bands, were computed as outcome measures. Frequency estimates were judged correct based on the following frequency bands (see Berry, Raynor & Knapp, 2003): Very Common = 10,001–100,000 (>10%); Common = 1001–10,000 (>1–10%); Uncommon = 101–1000 (>0.1–1%); Rare = 11–100 (>0.01–0.1%); Very Rare = 0–10 (0–0.01%). ANOVAs were performed with Framing and Familiarity as factors.

Results

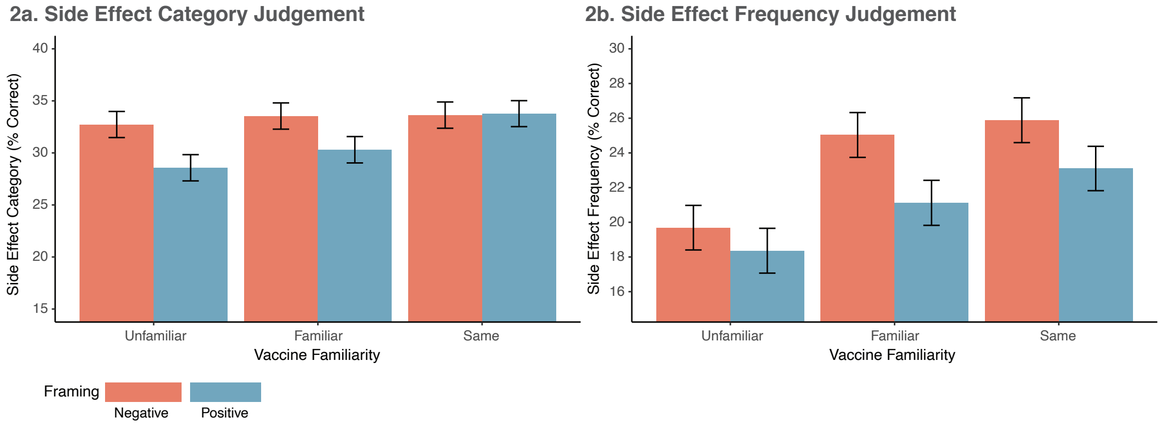

Supplementary Figure S2: Mean pearcentage of correct category (2a) and frequency (2b) judgements. All error bars represent +/- 1 SEM.

Verbal Prevalence Categories

As depicted in Supplementary Figure 2a, a main effect of Framing ($F(1, 1216)$=5.54, p=.019, *np2*=.005) was observed, while Familiarity neared significance ($F(2, 1216)$=2.98, p=.051, *np2*=.005). This was driven by Contrast1 only (Same vs. Other: $F(1, 1216)$=4.93, p=.027, *np2*=.004). While accuracy was generally low, fewer accurate classifications were made following Positive Framing (M=30.9%, *SEM*=0.73, 95% CIs [29.46, 32.31]), relative to Negative Framing (M=33.3%, *SEM*=0.73, 95% CIs [31.87, 43.73]). While the reduction in accuracy associated with positive, relative to negative, framing appeared to decrease with familiarity, the Framing by Contrast1(Same vs. Other) interaction did not reach statistical significance ($F(1, 1216)$=3.11, p=.078, *np2*=.003).

Verbal Prevalence Categories: Statistical Models

Overall_CategoryPercent = percentage of correctly identified categories

Main Effects Model

	Sum Sq	Df	F value	Pr(>F)
(Intercept)	1258379.599	1	3900.3990	0.0000
Framing	1786.274	1	5.5366	0.0188
Familiarity	1923.618	2	2.9812	0.0511
Framing:Familiarity	1047.111	2	1.6228	0.1978
Residuals	392316.166	1216	NA	NA

	eta.sq	eta.sq.part
Framing	0.0045	0.0045
Familiarity	0.0048	0.0049
Framing:Familiarity	0.0026	0.0027

Model with pre-registered contrasts for Vaccine Familiarity

	Sum Sq	Df	F value	Pr(>F)
(Intercept)	1258379.5992	1	3900.3990	0.0000
Framing	1786.2735	1	5.5366	0.0188
cont1	1590.3548	1	4.9294	0.0266
cont2	329.1191	1	1.0201	0.3127
Framing:cont1	1003.2909	1	3.1097	0.0781
Framing:cont2	42.6316	1	0.1321	0.7163
Residuals	392316.1662	1216	NA	NA

	eta.sq	eta.sq.part
Framing	0.0045	0.0045
cont1	0.0040	0.0040
cont2	0.0008	0.0008
Framing:cont1	0.0025	0.0026
Framing:cont2	0.0001	0.0001

Frequency Estimates

A main effect of Framing ($F(1, 1216)=6.45$, $p=.011$, *np2*=.005) and Familiarity ($F(2, 1216)=9.71$, $p=.0001$, *np2*=.016) was observed. As depicted in Supplementary Figure 2b, accuracy was reduced with Positive Framing, but increased with Familiarity. Contrast1 (Same vs. Other: $F(1, 1216)=9.52$, $p=.002$, *np2*=.008) and Contrast2 (Familiar vs. Unfamiliar: $F(1, 1216)=9.84$, $p=.002$, *np2*=.008) reached significance. Overall, participants frequency estimates were less accurate following positive framing (M=20.7%, *SEM*=0.74, 95% CIs [19.40, 22.32]) relative to negative framing (M=23.54%, *SEM*=0.74, 95% CIs [22.08, 25.00]). Framing did not interact with the main effect of Familiarity, or the orthogonal contrasts (all *ps*>.30).

Neither Framing nor Familiarity had any effect on frequency estimates of side effects of 'unknown' frequency (all *ps*>.16). Those in the Positive Framing conditions estimated that these side effects would be experienced by 1,848/100,000 people on average, and those in the Negative Frame as 2,004/100,000.

Frequency Estimates: Statistical Models

Overall_FrequencyPercent = percentage of correctly estimated frequency brackets

Main Effects Model

	Sum Sq	Df	F value	Pr(>F)
(Intercept)	602115.9981	1	1777.6846	0.0000
Framing	2185.5526	1	6.4526	0.0112
Familiarity	6577.8779	2	9.7102	0.0001
Framing:Familiarity	340.6265	2	0.5028	0.6049
Residuals	411868.9225	1216	NA	NA

	eta.sq	eta.sq.part
Framing	0.0052	0.0053
Familiarity	0.0156	0.0157
Framing:Familiarity	0.0008	0.0008

Model with pre-registered contrasts for Vaccine Familiarity

	Sum Sq	Df	F value	Pr(>F)
(Intercept)	602115.9981	1	1777.6846	0.0000
Framing	2185.5526	1	6.4526	0.0112
cont1	3225.6804	1	9.5235	0.0021
cont2	3333.4302	1	9.8416	0.0017
Framing:cont1	1.8231	1	0.0054	0.9415
Framing:cont2	338.6588	1	0.9999	0.3175
Residuals	411868.9225	1216	NA	NA

	eta.sq	eta.sq.part
Framing	0.0052	0.0053
cont1	0.0077	0.0078
cont2	0.0079	0.0080
Framing:cont1	0.0000	0.0000
Framing:cont2	0.0008	0.0008

Frequency estimates for side effect of 'unknown prevalence'

Main Effects Model

	Sum Sq	Df	F value	Pr(>F)
(Intercept)	4533390495	1	29.5304	0.0000
Framing	7457785	1	0.0486	0.8256
Familiarity	537592293	2	1.7509	0.1740
Framing:Familiarity	119664905	2	0.3897	0.6773
Residuals	186675244185	1216	NA	NA

	eta.sq	eta.sq.part
Framing	0.0000	0.0000
Familiarity	0.0029	0.0029
Framing:Familiarity	0.0006	0.0006

S1.2: Wording used for the Positive and Negative Attribute Frame

Negative Attribute Frame (Standard EU Wording)	Positive Attribute Frame	Positive Attribute Frame (Accompanying Infographic)
**Very common** (may affect more than 1 in 10 people)	**Very common** (9 in 10 or fewer people may ***not be affected***)	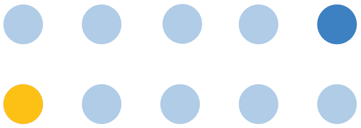 **Very Common:** 9 in 10 or fewer **not affected** (light to dark blue circles)
**Common** (may affect up to 1 in 10 people)	**Common** (90 in 100 or more people may ***not be affected***)	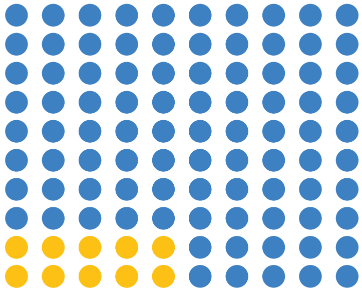 **Common:** 90 in 100 (blue circles) or more **not affected**
**Uncommon** (may affect up to 1 in 100 people)	**Uncommon** (990 in 1,000 or more people may ***not be affected***)	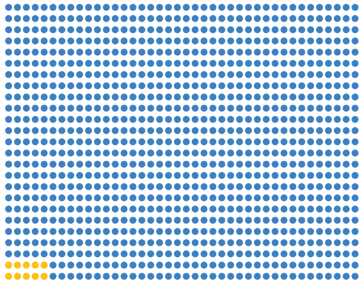 **Uncommon:** 990 in 1,000 (blue circles) or more **not affected**
**Rare** (may affect up to 1 in 1,000 people)	**Rare** (9,990 in 10,000 or more people may ***not be affected***)	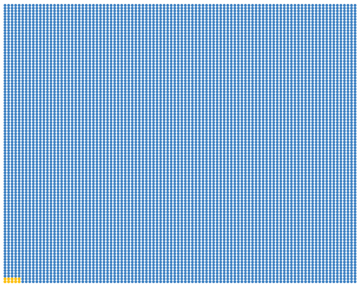 **Rare:** 9,990 in 10,000 (blue circles) or more **not affected**
**Very Rare** (may affect up to 1 in 10,000 people)	**Very Rare** (99,990 in 100,000 or more people may ***not be affected***)	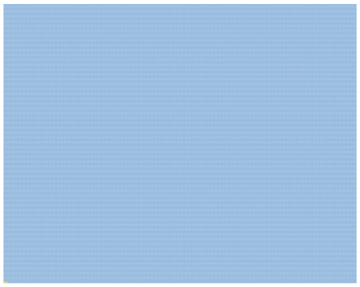 **Very Rare:** 99,990 in 100,000 (blue circles) or more **not affected**

S1.3: Patient Information Leaflets (PILs)

The following pages contain the PILs used to present side effect information.

High fidelity versions of these documents were embedded in Qualtrics.

Negative PIL (AstraZeneca Vaccine)

Package leaflet: Information for the user

Vaxzevria suspension for injection
COVID-19 Vaccine (ChAdOx1-S [recombinant])

▼This medicine is subject to additional monitoring. This will allow quick identification of new safety information. You can help by reporting any side effects you may get.

Read all of this leaflet carefully before the vaccine is given because it contains important information for you.

What Vaxzevria is and what it is used for

Vaxzevria is used for preventing COVID-19 caused by the SARS-CoV-2 virus.

Vaxzevria is given to adults aged 18 years and older.

The vaccine causes the immune system (the body's natural defences) to produce antibodies and specialised white blood cells that work against the virus, so giving protection against COVID-19.
None of the ingredients in this vaccine can cause COVID-19

Possible side effects

Like all medicines, this vaccine can cause side effects, although not everybody gets them.

The following side effects may occur with Vaxzevria:

Very Common (may affect more than 1 in 10 people)

- tenderness, pain, warmth, itching, or bruising where the injection is given
- feeling tired (fatigue) or generally feeling unwell
- chills or feeling feverish
- headache
- feeling sick (nausea)
- joint pain or muscle ache

Common (may affect up to 1 in 10 people)

- swelling or redness where the injection is given
- fever (≥38°C)
- being sick (vomiting) or diarrhoea
- low level of blood platelets
- pain in legs or arms
- flu-like symptoms, such as high temperature, sore throat, runny nose, cough and chills
- physical weakness or lack of energy

Uncommon (may affect up to 1 in 100 people)

- sleepiness or feeling dizzy
- abdominal pain or decreased appetite
- enlarged lymph nodes
- excessive sweating, itchy skin, rash or hives
- sleepiness or deep unresponsiveness and inactivity

Very Rare (may affect up to 1 in 10,000 people)

- blood clots often in unusual locations (e.g. brain, bowel, liver, spleen) in combination with low level of blood platelets
- serious nerve inflammation, which may cause paralysis and difficulty breathing (Guillain-Barré syndrome [GBS])

Not known (cannot be estimated from the available data)

- severe allergic reaction (anaphylaxis)
- hypersensitivity
- rapid swelling under the skin in areas such as the face, lips, mouth and throat (which may cause difficulty in swallowing or breathing)
- capillary leak syndrome (a condition causing fluid leakage from small blood vessels)

Postive PIL (AstraZeneca Vaccine)

Package leaflet: Information for the user

Vaxzevria suspension for injection
COVID-19 Vaccine (ChAdOx1-S [recombinant])

▼This medicine is subject to additional monitoring. This will allow quick identification of new safety information. You can help by reporting any side effects you may get.

Read all of this leaflet carefully before the vaccine is given because it contains important information for you.

What Vaxzevria is and what it is used for

Vaxzevria is used for preventing COVID-19 caused by the SARS-CoV-2 virus.

Vaxzevria is given to adults aged 18 years and older.

The vaccine causes the immune system (the body's natural defences) to produce antibodies and specialised white blood cells that work against the virus, so giving protection against COVID-19.
None of the ingredients in this vaccine can cause COVID-19

Possible side effects

Like all medicines, this vaccine can cause side effects, although not everybody gets them.

The following side effects may occur with Vaxzevria:

In the images below:
Blue circles represent people **not affected**
Yellow circles represent people who **are affected**

Very Common (9 in 10 or fewer people may ***not be affected***)

- tenderness, pain, warmth, itching, or bruising where the injection is given
- feeling tired (fatigue) or generally feeling unwell
- chills or feeling feverish
- headache
- feeling sick (nausea)
- joint pain or muscle ache

Very Common: 9 in 10 or fewer **not affected** (light to dark blue circles)

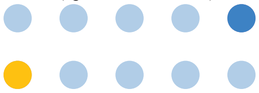

Common (90 in 100 or more people may ***not be affected***)

- swelling or redness where the injection is given
- fever (≥38°C)
- being sick (vomiting) or diarrhoea
- low level of blood platelets
- pain in legs or arms
- flu-like symptoms, such as high temperature, sore throat, runny nose, cough and chills
- physical weakness or lack of energy

Common: 90 in 100 (blue circles) or more **not affected**

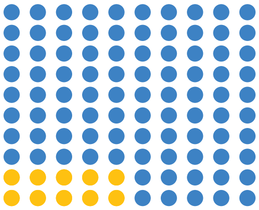

Uncommon (990 in 1,000 or more people may ***not be affected***)

- sleepiness or feeling dizzy
- abdominal pain or decreased appetite
- enlarged lymph nodes
- excessive sweating, itchy skin, rash or hives
- sleepiness or deep unresponsiveness and inactivity

Uncommon: 990 in 1,000 (blue circles) or more **not affected**

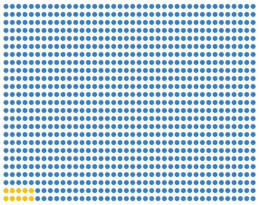

Very Rare (99,990 in 100,000 or more people may ***not be affected***)

- blood clots often in unusual locations (e.g. brain, bowel, liver, spleen) in combination with low level of blood platelets
- serious nerve inflammation, which may cause paralysis and difficulty breathing (Guillain-Barré syndrome [GBS])

Very Rare: 99,990 in 100,000 (blue circles) or more **not affected**

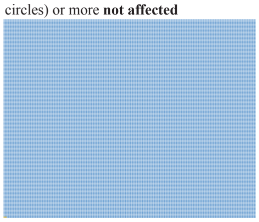

Not known (cannot be estimated from the available data)

- severe allergic reaction (anaphylaxis)
- hypersensitivity
- rapid swelling under the skin in areas such as the face, lips, mouth and throat (which may cause difficulty in swallowing or breathing)
- capillary leak syndrome (a condition causing fluid leakage from small blood vessels)

Negative PIL (Pfizer Vaccine)

Package leaflet: Information for the user

Comirnaty concentrate for dispersion for injection
COVID-19 mRNA Vaccine (nucleoside modified)

▼This medicine is subject to additional monitoring. This will allow quick identification of new safety information. You can help by reporting any side effects you may get.

Read all of this leaflet carefully before you receive this vaccine because it contains important information for you.

What Comirnaty is and what it is used for

Comirnaty is a vaccine used for preventing COVID-19 caused by SARS-CoV-2 virus.

Comirnaty is given to adults and adolescents from 12 years of age and older.

The vaccine causes the immune system (the body's natural defences) to produce antibodies and blood cells that work against the virus, so giving protection against COVID-19.

As Comirnaty does not contain the virus to produce immunity, it cannot give you COVID-19.

Possible side effects

Like all vaccines, Comirnaty can cause side effects, although not everybody gets them.

The following side effects may occur with Comirnaty:

Very common side effects (may affect more than 1 in 10 people)

- injection site: pain, swelling
- tiredness
- headache
- muscle pain
- chills
- joint pain
- diarrhoea
- fever

Some of these side effects were slightly more frequent in adolescents 12 to 15 years than in adults.

Common side effects (may affect up to 1 in 10 people)

- injection site redness
- nausea
- vomiting

Uncommon side effects (may affect up to 1 in 100 people)

- enlarged lymph nodes
- feeling unwell
- arm pain
- insomnia
- injection site itching
- allergic reactions such as rash or itching

Rare side effects (may affect up to 1 in 1,000 people)

- temporary one sided facial drooping
- allergic reactions such as hives or swelling of the face

Not known (cannot be estimated from the available data)

- severe allergic reaction
- inflammation of the heart muscle (myocarditis) or inflammation of the lining outside the heart (pericarditis) which can result in breathlessness, palpitations or chest pain
- extensive swelling of the vaccinated limb
- swelling of the face (swelling of the face may occur in patients who have had facialdermatological fillers)

Postive PIL (Pfizer Vaccine)

Package leaflet: Information for the user

Comirnaty concentrate for dispersion for injection
COVID-19 mRNA Vaccine (nucleoside modified)

▼This medicine is subject to additional monitoring. This will allow quick identification of new safety information. You can help by reporting any side effects you may get.

Read all of this leaflet carefully before you receive this vaccine because it contains important information for you.

What Comirnaty is and what it is used for

Comirnaty is a vaccine used for preventing COVID-19 caused by SARS-CoV-2 virus.

Comirnaty is given to adults and adolescents from 12 years of age and older.

The vaccine causes the immune system (the body's natural defences) to produce antibodies and blood cells that work against the virus, so giving protection against COVID-19.

As Comirnaty does not contain the virus to produce immunity, it cannot give you COVID-19.

Possible side effects

Like all vaccines, Comirnaty can cause side effects, although not everybody gets them.

The following side effects may occur with Comirnaty:

In the images below:
Blue circles represent people **not affected**
Yellow circles represent people who **are affected**

Very common side effects (9 in 10 or fewer people may ***not be affected***)

- injection site: pain, swelling
- tiredness
- headache
- muscle pain
- chills
- joint pain
- diarrhoea
- fever

Very Common: 9 in 10 or fewer **not affected** (light to dark blue circles)

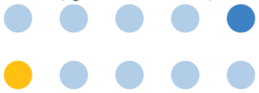

Some of these side effects were slightly more frequent in adolescents 12 to 15 years than in adults.

Common side effects (90 in 100 or more people may ***not be affected***)

- injection site redness
- nausea
- vomiting

Common: 90 in 100 (blue circles) or more **not affected**

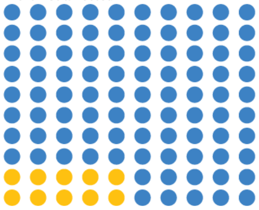

Uncommon side effects (990 in 1,000 or more people may ***not be affected***)

- enlarged lymph nodes
- feeling unwell
- arm pain
- insomnia
- injection site itching
- allergic reactions such as rash or itching

Uncommon: 990 in 1,000 (blue circles) or more **not affected**

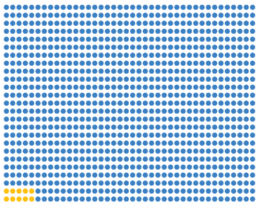

Rare side effects (9,990 in 10,000 or more people may ***not be affected***)

- temporary one sided facial drooping
- allergic reactions such as hives or swelling of the face

Rare: 9,990 in 10,000 (blue circles) or more **not affected**

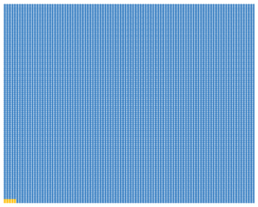

Not known (cannot be estimated from the available data)

- severe allergic reaction
- inflammation of the heart muscle (myocarditis) or inflammation of the lining outside the heart (pericarditis) which can result in breathlessness, palpitations or chest pain
- extensive swelling of the vaccinated limb
- swelling of the face (swelling of the face may occur in patients who have had facialdermatological fillers)

Negative PIL (Moderna Vaccine)

Package leaflet: Information for the user

Spikevax dispersion for injection
COVID-19 mRNA Vaccine (nucleoside modified)

▼This medicine is subject to additional monitoring. This will allow quick identification of new safety information. You can help by reporting any side effects you may get.

Read all of this leaflet carefully before you receive this vaccine because it contains important information for you.

What Spikevax is and what it is used for

Spikevax is a vaccine used to prevent COVID-19 caused by SARS-CoV-2. It is given to adults and children aged 12 years and older. The active substance in Spikevax is mRNA encoding the SARS-CoV-2 Spike protein. The mRNA is embedded in SM-102 lipid nanoparticles.
As Spikevax does not contain the virus, it cannot give you COVID-19.

How the vaccine works
Spikevax stimulates the body's natural defences (immune system). The vaccine works by causing the body to produce protection (antibodies) against the virus that causes COVID-19.

Possible side effects

Like all medicines, this vaccine can cause side effects, although not everybody gets them.

The following side effects may occur with Spikevax:

Very common (may affect more than 1 in 10 people)

- swelling/tenderness of the underarm glands
- headache
- nausea
- vomiting
- muscle ache, joint aches, and stiffness
- pain or swelling at the injection site
- feeling very tired
- chills
- fever

Common (may affect up to 1 in 10 people)

- rash
- rash, redness, or hives at the injection site (some of which may occur some time after the
- injection)

Uncommon (may affect up to 1 in 100 people)

- itchiness at the injection site

Rare (may affect up to 1 in 1,000 people)

- temporary one-sided facial drooping (Bell's palsy)
- swelling of the face (Swelling of the face may occur in patients who have had facial cosmetic
- injections.)
- dizziness
- decreased sense of touch or sensation

Frequency unknown (cannot be estimated from the available data)

- severe allergic reactions with breathing difficulties (anaphylaxis)
- reaction of increased sensitivity or intolerance by the immune system (hypersensitivity)
- inflammation of the heart muscle (myocarditis) or inflammation of the lining outside the heart (pericarditis) which can result in breathlessness, palpitations or chest pain.

Postive PIL (Moderna Vaccine)

Package leaflet: Information for the user

Spikevax dispersion for injection
COVID-19 mRNA Vaccine (nucleoside modified)

▼This medicine is subject to additional monitoring. This will allow quick identification of new safety information. You can help by reporting any side effects you may get.

Read all of this leaflet carefully before you receive this vaccine because it contains important information for you.

What Spikevax is and what it is used for

Spikevax is a vaccine used to prevent COVID-19 caused by SARS-CoV-2. It is given to adults and children aged 12 years and older. The active substance in Spikevax is mRNA encoding the SARS-CoV-2 Spike protein. The mRNA is embedded in SM-102 lipid nanoparticles.
As Spikevax does not contain the virus, it cannot give you COVID-19.

How the vaccine works
Spikevax stimulates the body's natural defences (immune system). The vaccine works by causing the body to produce protection (antibodies) against the virus that causes COVID-19.

Possible side effects

Like all medicines, this vaccine can cause side effects, although not everybody gets them.

The following side effects may occur with Spikevax:

In the images below:
Blue circles represent people **not affected**
Yellow circles represent people who **are affected**

Very common (9 in 10 or fewer people may ***not be affected***)

- swelling/tenderness of the underarm glands
- headache
- nausea
- vomiting
- muscle ache, joint aches, and stiffness
- pain or swelling at the injection site
- feeling very tired
- chills
- fever

Very Common: 9 in 10 or fewer **not affected** (light to dark blue circles)

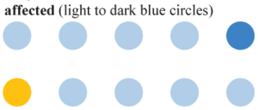

Common (90 in 100 or more people may ***not be affected***)

- rash
- rash, redness, or hives at the injection site (some of which may occur some time after the
- injection)

Common: 90 in 100 (blue circles) or more **not affected**

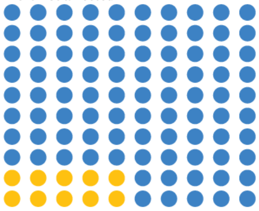

Uncommon (990 in 1,000 or more people may ***not be affected***)

- itchiness at the injection site

Uncommon: 990 in 1,000 (blue circles) or more **not affected**

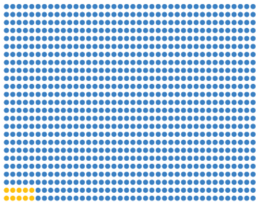

Rare (9,990 in 10,000 or more people may ***not be affected***)

- temporary one-sided facial drooping (Bell's palsy)
- swelling of the face (Swelling of the face may occur in patients who have had facial cosmetic
- injections.)
- dizziness
- decreased sense of touch or sensation

Rare: 9,990 in 10,000 (blue circles) or more **not affected**

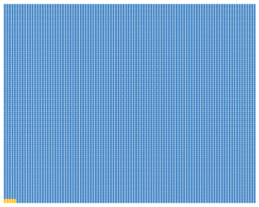

Frequency unknown (cannot be estimated from the available data)

- severe allergic reactions with breathing difficulties (anaphylaxis)
- reaction of increased sensitivity or intolerance by the immune system (hypersensitivity)
- inflammation of the heart muscle (myocarditis) or inflammation of the lining outside the heart (pericarditis) which can result in breathlessness, palpitations or chest pain.

S1.4: Pre-analysis data cleaning

Participants (N = 237) were excluded based on meeting one or more of the following pre-registered quality control checks:

1. Completed the study quicker than would be expected given a reasonable reading rate (8 minutes, 5th percentile of soft-launch data)
2. Failed to answer attention questions appropriately (e.g., place slider in X position)
3. Provided discrepant data during screening and the main survey regarding the COVID-19 vaccine they had previously received (i.e., AstraZeneca or Pfizer)
4. Identified as having experienced side effects to previous COVID-19 vaccines, then did not select any side effects
5. Spent more than 5 minutes on the 2-minute timed manipulation page without proceeding
6. Identified as having previously received a specific COVID-19 vaccine, then stated they had never heard of that vaccine

S1.5: Side effects presented from each PIL

The following presents the 14 side effects associated with each vaccine type (i.e., AstraZeneca, Pfizer, Moderna).

AstraZeneca Side Effects	
Category	***Side Effect ($N = 11$)***
Very Common	Injection site pain
Very Common	Tiredness (fatigue)
Very Common	Muscle ache/pain
Very Common	Chills
Common	Injection site redness
Common	Vomiting
Uncommon	Diarrhea
Uncommon	Enlarged lymph nodes (swelling of underarm glands)
Uncommon	Dizziness
Very Rare	Blood clots often in unusual locations (e.g. brain, bowel, liver, spleen) in combination with low level of blood platelets
Very Rare	Serious nerve inflammation, which may cause paralysis and difficulty breathing (Guillain-Barré syndrome)

AstraZeneca Unknown Side Effects	
Category	***Side Effect ($N = 3$)***
Not known	Severe allergic reaction (anaphylaxis)
Not known	hypersensitivity
Not known	capillary leak syndrome (a condition causing fluid leakage from small blood vessels)

Pfizer Side Effects	
**Category**	**Side Effect (N = 11)**
Very Common	Injection site pain
Very Common	Tiredness (fatigue)
Very Common	Muscle ache/pain
Very Common	Chills
Common	Injection site redness
Common	Vomiting
Uncommon	Diarrhea
Uncommon	Enlarged lymph nodes (swelling of underarm glands)
Uncommon	Insomnia
Rare	Temporary one-sided facial drooping
Rare	Swelling of the face

Pfizer Unknown Side Effects	
**Category**	**Side Effect (N = 3)**
Not known	Severe allergic reaction (anaphylaxis)
Not known	Inflammation of the heart (myocarditis or pericarditis)
Not known	Extensive swelling of the vaccinated limb

Moderna Side Effects	
**Category**	**Side Effect (N = 11)**
Very Common	Injection site pain
Very Common	Tiredness (fatigue)
Very Common	Muscle ache/pain
Very Common	Chills
Common	Injection site redness
Common	Rash
Common	Injection site hives
Uncommon	injection site itching
Rare	Temporary one-sided facial drooping
Rare	Swelling of the face
Rare	Decreased sense of touch or sensation

Moderna Unknown Side Effects	
**Category**	**Side Effect (N = 3)**
Not known	Severe allergic reaction (anaphylaxis)
Not known	Hypersensitivity
Not known	Inflammation of the heart (myocarditis or pericarditis)

S1.6: Full statistical model for the primary outcome

The following analysis includes interactions between the factors (Framing/Familiarity) and the planned covariate (Baseline Booster Intention)

Variables are:

- intent_fv_t2 (primary outcome: booster intention (intent) for the framed vaccine (fv) post-manipulation (t2))
- intent_fv_t1 (covariate: booster intention (intent) for the framed vaccine (fv) at baseline (t1))

Full model accounting for interactions with the covariate

	Sum Sq	Df	F value	Pr(>F)
(Intercept)	48462.8455	1	173.6635	0.0000
intent_fv_t1	543030.3892	1	1945.9146	0.0000
Framing	25.7614	1	0.0923	0.7613
Familiarity	4466.3893	2	8.0025	0.0004
intent_fv_t1:Framing	76.6206	1	0.2746	0.6004
intent_fv_t1:Familiarity	5867.6007	2	10.5131	0.0000
Framing:Familiarity	6002.1555	2	10.7542	0.0000
intent_fv_t1:Framing:Familiarity	4268.1448	2	7.6473	0.0005
Residuals	337664.7592	1210	NA	NA

	eta.sq	eta.sq.part
intent_fv_t1	0.5321	0.6166
Framing	0.0000	0.0001
Familiarity	0.0044	0.0131
intent_fv_t1:Framing	0.0001	0.0002
intent_fv_t1:Familiarity	0.0057	0.0171
Framing:Familiarity	0.0059	0.0175
intent_fv_t1:Framing:Familiarity	0.0042	0.0125

Full model with pre-registered contrasts for Vaccine Familiarity

- cont1 = Same Vaccine vs. Other Vaccines [Familiar and Unfamiliar combined]
- cont2 = Familiar Vaccine vs. Unfamiliar Vaccine

	Sum Sq	Df	F value	Pr(>F)
(Intercept)	48462.8455	1	173.6635	0.0000
intent_fv_t1	543030.3892	1	1945.9146	0.0000
Framing	25.7614	1	0.0923	0.7613
cont1	3134.4070	1	11.2319	0.0008
cont2	1335.9564	1	4.7873	0.0289
intent_fv_t1:Framing	76.6206	1	0.2746	0.6004
intent_fv_t1:cont1	4420.8055	1	15.8417	0.0001
Framing:cont1	1413.4722	1	5.0651	0.0246
intent_fv_t1:cont2	1553.5052	1	5.5669	0.0185
Framing:cont2	4593.6316	1	16.4610	0.0001
intent_fv_t1:Framing:cont1	1225.7388	1	4.3924	0.0363
intent_fv_t1:Framing:cont2	3122.1111	1	11.1879	0.0008
Residuals	337664.7592	1210	NA	NA

	eta.sq	eta.sq.part
intent_fv_t1	0.5321	0.6166
Framing	0.0000	0.0001
cont1	0.0031	0.0092
cont2	0.0013	0.0039
intent_fv_t1:Framing	0.0001	0.0002
intent_fv_t1:cont1	0.0043	0.0129
Framing:cont1	0.0014	0.0042
intent_fv_t1:cont2	0.0015	0.0046
Framing:cont2	0.0045	0.0134
intent_fv_t1:Framing:cont1	0.0012	0.0036
intent_fv_t1:Framing:cont2	0.0031	0.0092

Framing	Familiarity	emmean	SE	df	lower.CL	upper.CL
Negative Frame	Unfamiliar	77.1205	1.1951	1210	74.7758	79.4651
Positive Frame	Unfamiliar	80.8857	1.2080	1210	78.5157	83.2556
Negative Frame	Familiar	79.6068	1.1804	1210	77.2910	81.9225
Positive Frame	Familiar	79.1131	1.1893	1210	76.7799	81.4464
Negative Frame	Same	80.3603	1.2930	1210	77.8235	82.8971
Positive Frame	Same	78.5867	1.3920	1210	75.8557	81.3176

S1.7: Pre-registered ANCOVA (without interactions between factors and covariate)

The following presents the results from the pre-registered ANCOVA that did not account for interactions between the covariate (baseline booster intention) and the Framing/Familiarity factors.

Main Effects Model

	Sum Sq	Df	F value	Pr(>F)
(Intercept)	81587.3803	1	285.3356	0.0000
intent_fv_t1	607341.4178	1	2124.0559	0.0000
Framing	667.2802	1	2.3337	0.1269
Familiarity	820.2857	2	1.4344	0.2387
Framing:Familiarity	1987.1180	2	3.4748	0.0313
Residuals	347410.7352	1215	NA	NA

	eta.sq	eta.sq.part
intent_fv_t1	0.5951	0.6361
Framing	0.0007	0.0019
Familiarity	0.0008	0.0024
Framing:Familiarity	0.0019	0.0057

Model with pre-registered contrasts for Vaccine Familiarity

	Sum Sq	Df	F value	Pr(>F)
(Intercept)	81587.3803	1	285.3356	0.0000
intent_fv_t1	607341.4178	1	2124.0559	0.0000
Framing	667.2802	1	2.3337	0.1269
cont1	801.2642	1	2.8023	0.0944
cont2	16.7860	1	0.0587	0.8086
Framing:cont1	236.0587	1	0.8256	0.3637
Framing:cont2	1747.3457	1	6.1110	0.0136
Residuals	347410.7352	1215	NA	NA

	eta.sq	eta.sq.part
intent_fv_t1	0.5951	0.6361
Framing	0.0007	0.0019
cont1	0.0008	0.0023
cont2	0.0000	0.0000
Framing:cont1	0.0002	0.0007
Framing:cont2	0.0017	0.0050

Framing*Familiarity Means

Framing	Familiarity	emmean	SE	df	lower.CL	upper.CL
Negative Frame	Unfamiliar	77.0667	1.1868	1215	74.7383	79.3951
Positive Frame	Unfamiliar	82.0994	1.1939	1215	79.7572	84.4417
Negative Frame	Familiar	79.7128	1.1885	1215	77.3810	82.0445
Positive Frame	Familiar	78.8776	1.1952	1215	76.5327	81.2226
Negative Frame	Same	81.1027	1.1989	1215	78.7505	83.4549
Positive Frame	Same	81.3391	1.1946	1215	78.9955	83.6827

S1.8: The effect of positive framing on vaccine switches

Full model including previous vaccine type ("prevax_type")

	Sum Sq	Df	F value	Pr(>F)
(Intercept)	48152.2239	1	173.6703	0.0000
intent_fv_t1	466284.3106	1	1681.7448	0.0000
Framing	68.7237	1	0.2479	0.6187
Familiarity	4162.4448	2	7.5063	0.0006
prevax_type	1763.3901	1	6.3600	0.0118
intent_fv_t1:Framing	143.0262	1	0.5159	0.4728
intent_fv_t1:Familiarity	5531.5062	2	9.9752	0.0001
Framing:Familiarity	7062.0571	2	12.7353	0.0000
intent_fv_t1:prevax_type	945.5318	1	3.4102	0.0650
Framing:prevax_type	7.3229	1	0.0264	0.8709
Familiarity:prevax_type	207.0593	2	0.3734	0.6885
intent_fv_t1:Framing:Familiarity	5204.8395	2	9.3861	0.0001
intent_fv_t1:Framing:prevax_type	0.3171	1	0.0011	0.9730
intent_fv_t1:Familiarity:prevax_type	317.3683	2	0.5723	0.5644
Framing:Familiarity:prevax_type	1611.3401	2	2.9058	0.0551
intent_fv_t1:Framing:Familiarity:prevax_type	930.3705	2	1.6778	0.1872
Residuals	332160.1508	1198	NA	NA

	eta.sq	eta.sq.part
intent_fv_t1	0.4569	0.5840
Framing	0.0001	0.0002
Familiarity	0.0041	0.0124
prevax_type	0.0017	0.0053
intent_fv_t1:Framing	0.0001	0.0004
intent_fv_t1:Familiarity	0.0054	0.0164
Framing:Familiarity	0.0069	0.0208
intent_fv_t1:prevax_type	0.0009	0.0028
Framing:prevax_type	0.0000	0.0000
Familiarity:prevax_type	0.0002	0.0006
intent_fv_t1:Framing:Familiarity	0.0051	0.0154
intent_fv_t1:Framing:prevax_type	0.0000	0.0000
intent_fv_t1:Familiarity:prevax_type	0.0003	0.0010
Framing:Familiarity:prevax_type	0.0016	0.0048
intent_fv_t1:Framing:Familiarity:prevax_type	0.0009	0.0028

S1.9: Vaccine Switch - AstraZeneca to Pfizer and Moderna

	Sum Sq	Df	F value	Pr(>F)
(Intercept)	34492.1950	1	105.8593	0.0000
intent_fv_t1	139372.9781	1	427.7470	0.0000
Framing	11.5190	1	0.0354	0.8510
Familiarity	833.9037	1	2.5593	0.1104
intent_fv_t1:Framing	7.8985	1	0.0242	0.8764
intent_fv_t1:Familiarity	1172.9565	1	3.5999	0.0585
Framing:Familiarity	4799.7488	1	14.7308	0.0001
intent_fv_t1:Framing:Familiarity	3707.3265	1	11.3781	0.0008
Residuals	130983.8324	402	NA	NA

	eta.sq	eta.sq.part
intent_fv_t1	0.5019	0.5155
Framing	0.0000	0.0001
Familiarity	0.0030	0.0063
intent_fv_t1:Framing	0.0000	0.0001
intent_fv_t1:Familiarity	0.0042	0.0089
Framing:Familiarity	0.0173	0.0353
intent_fv_t1:Framing:Familiarity	0.0134	0.0275

S1.10: Vaccine Switch - Pfizer to Moderna

	Sum Sq	Df	F value	Pr(>F)
(Intercept)	26187.1494	1	59.7228	0.0000
intent__fv__t1	111498.8315	1	254.2860	0.0000
Framing	1744.2603	1	3.9780	0.0475
intent__fv__t1:Framing	791.0549	1	1.8041	0.1808
Residuals	86818.6664	198	NA	NA

	eta.sq	eta.sq.part
intent__fv__t1	0.5565	0.5622
Framing	0.0087	0.0197
intent__fv__t1:Framing	0.0039	0.0090

Model with pre-registered contrasts for Vaccine Familiarity

	Sum Sq	Df	F value	Pr(>F)
(Intercept)	4.533390e+09	1	29.5304	0.0000
Framing	7.457785e+06	1	0.0486	0.8256
cont1	2.443971e+08	1	1.5920	0.2073
cont2	2.947225e+08	1	1.9198	0.1661
Framing:cont1	5.626220e+04	1	0.0004	0.9847
Framing:cont2	1.195929e+08	1	0.7790	0.3776
Residuals	1.866752e+11	1216	NA	NA

	eta.sq	eta.sq.part
Framing	0.0000	0.0000
cont1	0.0013	0.0013
cont2	0.0016	0.0016
Framing:cont1	0.0000	0.0000
Framing:cont2	0.0006	0.0006

Mean estimates * Framing

Framing	emmean	SE	df	lower.CL	upper.CL
Negative Frame	2004.294	501.2572	1216	1020.8692	2987.719
Positive Frame	1848.045	501.2893	1216	864.5571	2831.533

S1.11: Exploratory Analysis

Post-hoc Familiarity * Framing * Baseline interactions among those with scores above and below the mean of baseline booster intention

Side Effect Severity

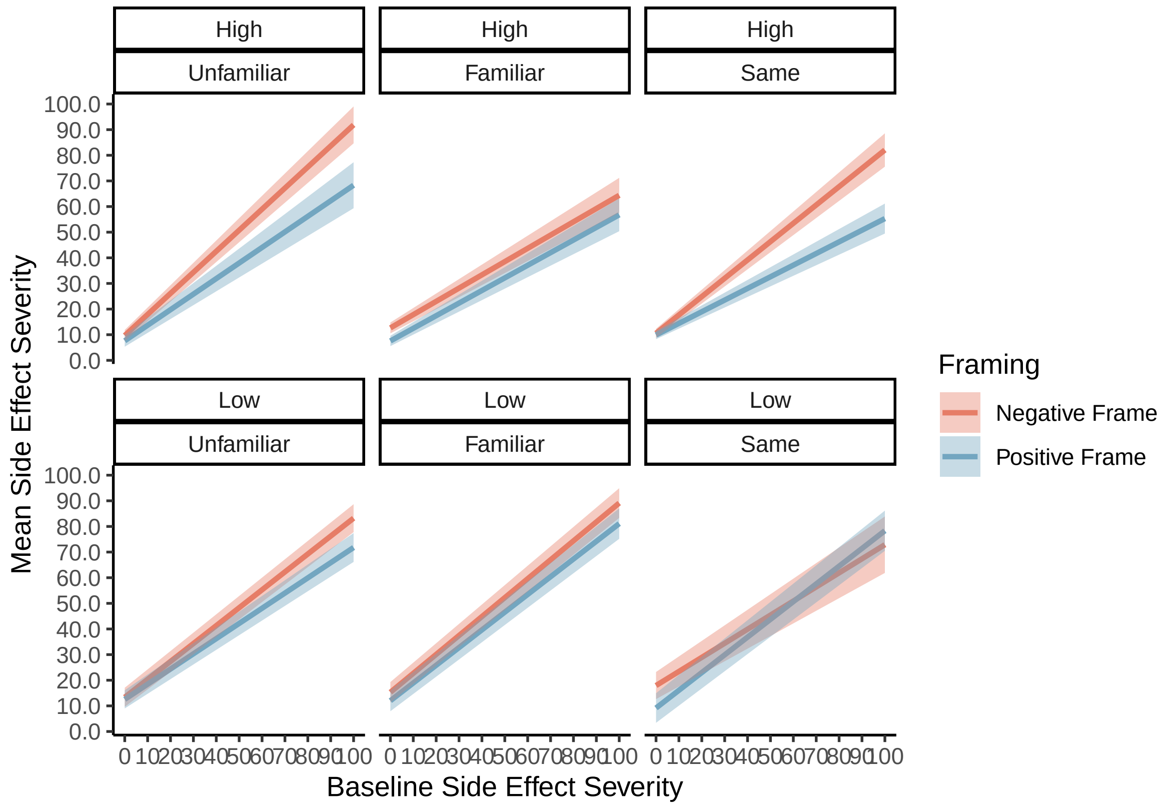

Booster Acceptance: Satisfaction and Happiness

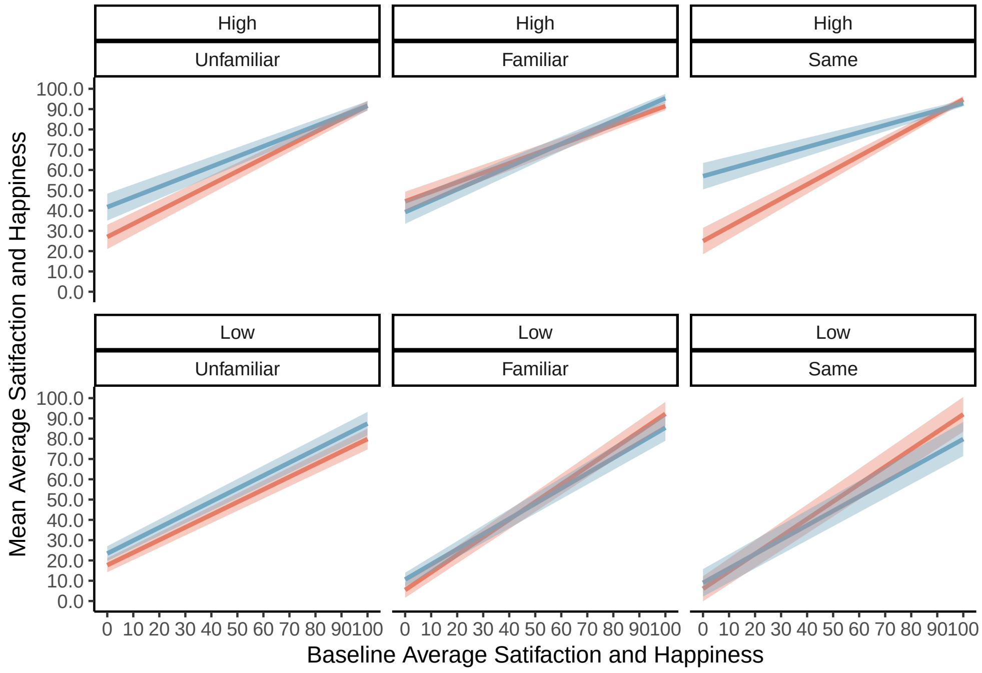

Booster Acceptance: Anxiety

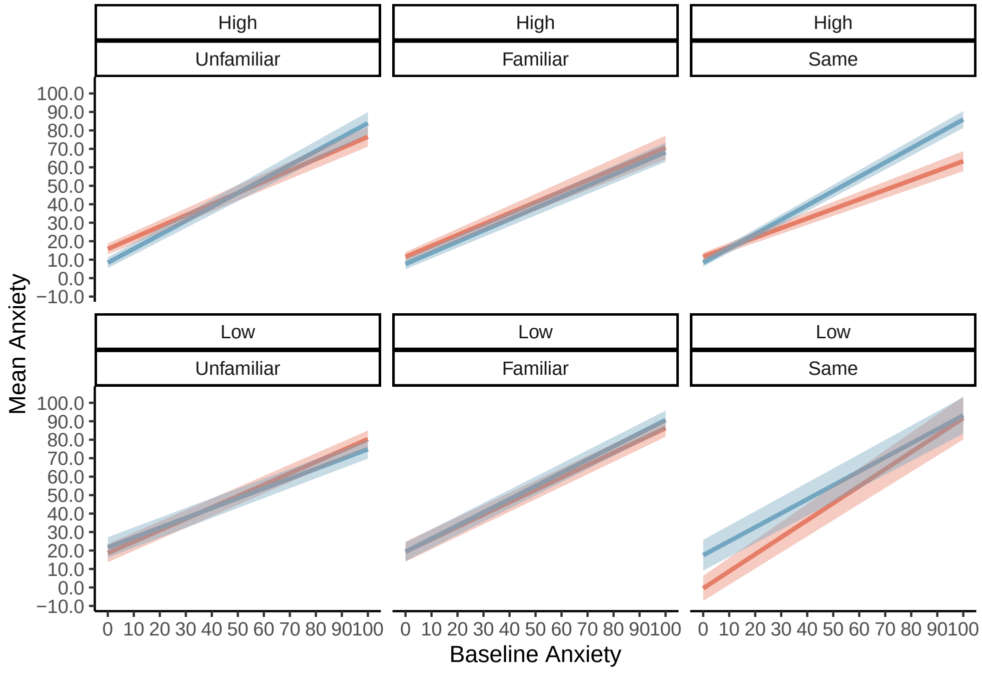

Supplement: Supplementary file 1 [file vaccines-10-00962-s001.zip › vaccines-1748228-Supplementary.pdf]
